# Treatment and outcome of Ganglioneuroma and Ganglioneuroblastoma intermixed

**DOI:** 10.1186/s12885-016-2513-9

**Published:** 2016-07-27

**Authors:** Boris Decarolis, Thorsten Simon, Barbara Krug, Ivo Leuschner, Christian Vokuhl, Peter Kaatsch, Dietrich von Schweinitz, Thomas Klingebiel, Ingo Mueller, Lothar Schweigerer, Frank Berthold, Barbara Hero

**Affiliations:** 1 Department of Pediatric Hematology and Oncology, Children’s Hospital, University of Cologne, Cologne, Germany; 2 Department of Radiology, University of Cologne, Cologne, Germany; 3 Department of Pathology, University of Kiel, Kiel, Germany; 4 German Childhood Cancer Registry, University of Mainz, Mainz, Germany; 5 Department of Pediatric Surgery, Dr von Hauner Children’s Hospital, Ludwig-Maximilians-University Munich, Munich, Germany; 6 Clinic for Pediatric Hematology and Oncology, Johann Wolfgang Goethe-University, Frankfurt, Germany; 7 Department of Pediatric Hematology and Oncology, University Medical Center Hamburg-Eppendorf, Hamburg-Eppendorf, Germany; 8 Clinic for Pediatrics, Helios Klinikum Berlin-Buch, Berlin-Buch, Germany

**Keywords:** Ganglioneuroma, Ganglioneuroblastoma intermixed, Therapy, Surgery, Subtotal resection, Treatment, Residual tumor

## Abstract

**Background:**

Ganglioneuroma (GN) and ganglioneuroblastoma intermixed (GNBI) are mature variants of neuroblastic tumors (NT). It is still discussed whether incomplete resection of GN/GNBI impairs the outcome of patients.

**Methods:**

Clinical characteristics and outcome of localized GN/GNBI were retrospectively compared to localized neuroblastoma (NB) and ganglioneuroblastoma-nodular (GNBN) registered in the German neuroblastoma trials between 2000 and 2010.

**Results:**

Of 808 consecutive localized NT, 162 (20 %) were classified as GN and 55 (7 %) as GNBI. GN/GNBI patients presented more often with stage 1 disease (68 % vs. 37 %, p < 0.001), less frequently with adrenal tumors (31 % vs. 43 %, p = 0.001) and positive mIBG-uptake (34 % vs. 90 %, p < 0.001), and had less often elevated urine catecholamine metabolites (homovanillic acid 39 % vs. 62 %, p < 0.001, vanillylmandelic acid 27 % vs. 64 %, p < 0.001). Median age at diagnosis increased with grade of differentiation (NB/GNBN: 9; GNBI: 61; GN-maturing: 71; GN-mature: 125 months, p < 0.001). Complete tumor resection was achieved at diagnosis in 70 % of 162 GN and 67 % of 55 GNBI, and after 4 to 32 months of observation in 4 GN (2 %) and 5 GNBI (9 %). Eleven patients received chemotherapy without substantial effect. Fifty-five residual tumors (42 GN, 13 GNBI) are currently under observation (median: 44 months). Five patients (3 GN, 2 GNBI) showed local progression; all had tumor residuals > 2 cm. No progression occurred after subtotal resection. Two patients died of treatment, none of tumor progression.

**Conclusions:**

GN/GNBI account for one quarter of localized NT and differ from immature tumors in their clinical features. Chemotherapy is not effective. Subtotal resection appears to be a sufficient treatment.

**Trial registration:**

ClinicalTrials.gov identifiers - NB97 (NCT00017225; registered June 6, 2001); NB2004 (NCT00410631; registered December 11, 2006)

**Electronic supplementary material:**

The online version of this article (doi:10.1186/s12885-016-2513-9) contains supplementary material, which is available to authorized users.

## Background

Neuroblastic tumors (NT) are the most common extra-cranial solid tumors in childhood [1] and include neuroblastoma, ganglioneuroblastoma (nodular or intermixed), and ganglioneuroma. They arise from the neural crest and range from immature, undifferentiated to mature, differentiated tumors. According to the International Neuroblastoma Pathology Classification (INPC) [2], ganglioneuroblastoma intermixed (GNBI) and ganglioneuroma (GN) represent the mature end of this range [3]. In this system, GN maturing has been defined as a “link” between GN and GNBI.

Ganglioneuroma has been first described more than 150 years ago [4]. A variety of case reports on GN have been published [4–14], ranging from patients with symptoms due to huge tumor masses [10] to speculations about malignant transformation and dedifferentiation into neuroblastoma [7, 8]. GN is generally considered a benign tumor that is treated by surgery alone. However, ganglioneuroblastoma intermixed (GNBI) is widely seen as a malignant entity and – depending on stage – treated with multimodal therapy. Case reports on GNBI are rarer [15, 16].

Only four larger series of GN and / or GNBI in pediatric patients have been reported [17–20]. In a previous analysis of our group, we assessed metabolic and clinical features of GN and demonstrated that a relevant proportion of GN shows mIBG uptake and elevated catecholamine metabolites in urine [17]. Furthermore, while complete resection was widely considered the standard treatment for GN, this analysis suggested that incomplete resection might be sufficient for the treatment of GN [17]. This was supported by De Bernardi et al who also suggested a more cautious surgical approach to localized GNBI and proposed that GN and GNBI show similar clinical behavior [18]. Analyses on the subgroups of GN maturing and GNBI by Cohn et al [19] and Okamatsu et al [20] supported this conclusion.

In this study, we retrospectively analyzed patients who were diagnosed with GN or GNBI in the last decade. We focused on localized stages as the typical presentations of GN and GNBI as metastatic disease is extremely rare in mature NT. Clinical features and course of GN and GNBI were compared to the group of immature localized NT. A special focus of our analysis was the outcome of patients with macroscopic tumor residuals in order to explore whether incomplete tumor resection is sufficient for the treatment of GN and GNBI.

## Methods

### Patients and parameters

The German neuroblastoma trials prospectively register all patients diagnosed in Germany with NT, including GN since mid of the 1990’s. The German neuroblastoma trials NB97 and NB2004 were approved by the ethical committee of the University of Cologne.

For this analysis, patients were included that met the following criteria: (a) registration to the German neuroblastoma trial office with written informed consent to participate (given by the patients or their parents / guardians for patients under 18 years of age), (b) diagnosis between January 1, 2000 and December 31, 2009 with localized neuroblastic tumor, (c) age at diagnosis 21 years or younger, (d) central histological review and classification according to INPC criteria [2], (e) diagnosis of GNBI or GN, histologically verified prior to any cytotoxic treatment. Patients with immature tumors (neuroblastoma (NB) and ganglioneuroblastoma nodular (GNBN)) that met the criteria (a) – (d) served as control. None of the patients has been included in the publication of Geoerger et al [17], while some of the patients were included in the international series published by Cohn et al [19].

Biological and clinical features and outcome were compared between mature and immature NT as well as between GN and GNBI. Tumor stage was classified according to the International Neuroblastoma Staging System (INSS) [21]. Status of MYCN oncogene and of the short arm of chromosome 1 was analyzed if a sufficient number of neuroblasts and/or ganglion cells could be analyzed in the available tumor material [22].

### Treatment

For patients with GN, tumor resection without any cytotoxic treatment was recommended. Patients with NB, GNBN and GNBI were treated according to the risk stratified GPOH neuroblastoma trials NB97 and NB2004. Treatment ranged from observation to intense multimodal treatment depending on tumor stage and molecular markers. Histology was not used for treatment stratification [23–25].

For this analysis, surgical tumor removal within 3 months after diagnosis was defined as initial surgery. Operations performed after this period were defined as delayed surgery.

### Radiology

Residual tumors were radiologically classified as minor or major residuals, defined by a maximum diameter of 2 cm in any extension in magnetic resonance imaging (MRI) as reported by local physician / radiologist. Tumor volume was calculated by the formula length * width * height / 2.

MRI series were centrally reviewed by the reference radiologist (B.K.) according to International Neuroblastoma Response Criteria (INRC) [21] and with respect to imaging quality for all patients with GN and GNBI with (suspected) tumor progression.

### Statistical analysis

Clinical features were analyzed using descriptive statistics. Differences between the groups were evaluated using the two-tailed χ^2^- test, Fisher’s exact test, Kruskal-Wallis-test, and the Mann-Whitney U test, whichever appropriate. Event free survival (EFS) and overall survival (OS) curves were generated using the Kaplan-Meier method [26] and compared by log-rank test [27]. Relapse, progression, and death of any reason were regarded as events.

## Results

### Patient cohort

Between January 1, 2000, and December 31, 2009, 1568 patients were registered in the German neuroblastoma trials NB97 and NB2004. 884 patients (56.4 %) had localized tumors of which 808 patients met the inclusion criteria as described above. About one quarter showed a mature histology. In detail, 162 of 808 tumors (20.0 %) were classified as GN. The vast majority (n = 144, 88.9 %) of GN were subclassified as maturing subtype, only 18 GN (11.1 %) as mature subtype. Fifty-five of 808 tumors (6.8 %) were GNBI.

### Clinical features (Table 1)

**Table 1:** Clinical features of the study cohort and the control group

	GN (n = 162)	p^1^	GNBI (n = 55)	GN / GNBI (n=217)	**p** ^**2**^	**NB / GNBN** **(n = 591)**
Localization						
Adrenal	51/161	0.613^*^	15/55	66/216	**0.001** ^*^	256/591
	31.7 %		27.3 %	30.6 %		43.3 %
Abdomino-pelvic	67/161		21/55	88/216		211/591
	41.6 %		38.2 %	40.7%		35.7 %
Thoraco-cervical	43/161		19/55	62/216		123/591
	26.7 %		34.5 %	28.7 %		20.8 %
INSS-stage						
Stage 1	112/160	0.404^**^	35/55	147/215	**<0.001** ^**^	216/591
	70.0 %		63.6 %	68.4%		36.5 %
Stage 2	30/160		16/55	46/215		201/591
	18.8 %		29.1 %	21.4%		34.0 %
Stage 3	18/160		4/55	22/215		174/591
	11.3 %		7.3 %	10.2%		29.4 %
Intraspinal tumor	16/162	0.223	9/55	25/217	0.212	89/591
	9.9 %		16.4 %	11.5 %		15.1 %
Tumor Volume (median; range)	75 ml (1.6 – 1100)	0.228	56.9 ml (1.5 – 871.9)	70.8 ml (1.5 – 1100)	**0.001**	49.5 ml (0.6 – 2300)
Positive mIBG-uptake	27/110	**<0.001**	22/36	49/146	**<0.001**	398/444
	24.5 %		61.1 %	33.6 %		89.6 %
HVA elevated	45/142	**0.001**	27/44	72/186	**<0.001**	327/531
	31.7 %		61.4 %	38.7 %		61.6 %
Elevation of HVA above upper limit (median; range)	0.98x (0.22 - 8.1)	0.137	1.46x (0.18 – 7.87)	1.1x (0.18 – 8.1)	**0.029**	1.5x (0.25 – 29)
VMA elevated	28/145	**<0.001**	23/47	51/192	**<0.001**	345/538
	19.3 %		48.9 %	26.6%		64.1 %
Elevation of VMA above upper limit (median; range)	0.71x (0.17 – 16.1)	0.061	1.3x (0.21 – 6.7)	0.9x (0.17 – 16.1)	**<0.001**	2.1x (0.24 – 40.3)
NSE elevated	50/124	0.150	22/41	72/165	**<0.001**	372/487
	40.3 %		53.7 %	43.6 %		76.4 %
NSE level (median; range)	18.3 ng/ml (4.9 – 49.7)	0.706	20.0 ng/ml (9.5 – 49.0)	19.0 ng/ml (4.9 – 49.7)	**<0.001**	37.2 ng/ml (7 – 2054)
Diagnosis by chance	70/161	0.163	30/55	100/216	0.177	306/591
	43.5 %		54,5 %	46.3 %		51.8 %

GN = Ganglioneuroma, GNBI = Ganglioneuroblastoma intermixed, NB = Neuroblastoma, GNBN = Ganglioneuroblastoma nodular,

INSS = International Neuroblastoma Staging System, HVA = homovanillic acid, VMA = vanillylmandelic acid, NSE = neuron specific enolase,

^*^ = adrenal vs. non-adrenal, ^**^ = stage 1 vs. stage 2/3, p^1^ = p-value GN vs. GNBI, p^2^ = p-value GN/GNBI vs. NB/GNBN, bold *p*-values = statistically significant

Patients with differentiated tumors (GN/GNBI) presented less frequently with adrenal tumors (30.6 % vs. 43.3 %, p = 0.001), showed less often positive mIBG-uptake (33.6 % vs. 89.6 %, p < 0.001) and less frequently elevated urine catecholamine metabolites (homovanillic acid 38.7 % vs. 61.6 %, p < 0.001, vanillylmandelic acid 26.6 % vs. 64.1 %, p < 0.001) than immature NT (NB/GNBN). Moreover, GN showed less often positive mIBG-uptake and elevated urine catecholamine metabolites than GNBI.

**Fig. 1:**
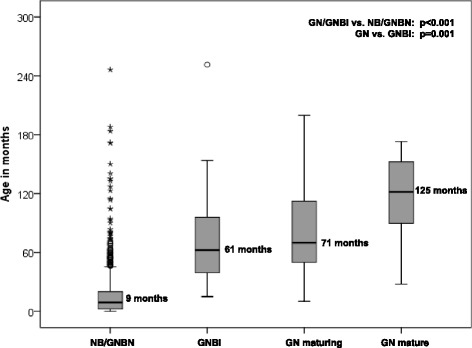
Grade of differentiation in relation to median age at diagnosis. NB = neuroblastoma, GNBN = ganglioneuroblastoma nodular, GNBI = ganglioneuroblastoma intermixed, GN = ganglioneuroma

Of interest, median age at diagnosis increased with the grade of neuroblastic differentiation as impressively shown in Fig. 1. Median tumor volume at diagnosis was also larger for GN/GNBI compared to NB/GNBN (70.8 ml vs. 49.5 ml, p = 0.001). Nevertheless, patients with GN/GNBI had more often stage 1 disease (68.4 % vs. 36.5 %, p < 0.001) than patients with immature NT. Nonetheless, 10.2 % of the differentiated tumors were stage 3 and 11.5 % showed intraspinal involvement.

Diagnosis of localized NT was made by routine check-ups or visits to the doctor for other reasons in 50 % of all cases with no significant difference between mature and immature tumors. As reported by local clinics, most frequent symptoms leading to diagnosis were pain (GN 34.2 %, GNBI 14.5 %, NB/GNBN 13.6 %, p < 0.001), palpable tumor mass (GN 9.3 %, GNBI 12.7 %, NB/GNBN 18.0 %, p = 0.022) and reduced general condition (GN 6.2 %, GNBI 7.3 %, NB/GNBN 15.8 %, p = 0.003).

No amplification of MYCN was detected in 90 children with GN and 53 patients with GNBI that were analyzed. Status of 1p was normal in 31 GN analyzed, while one out of 24 GNBI showed imbalance for 1p.

### Surgery (Fig. 2)

**Fig. 2:**
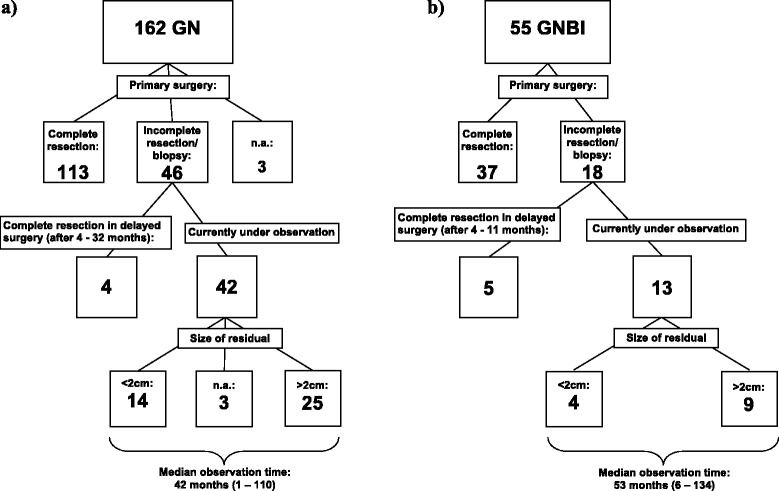
Extent of initial surgeries and size of residual tumors currently under observation. GNBI = ganglioneuroblastoma intermixed, GN = Ganglioneuroma, n.a. = data not available. **a**) Surgeries of patients with Ganglioneuroma, **b**) Surgeries of patients with Ganglioneurblastoma

For 159 patients with GN, data on extent of initial surgery was available. Complete tumor resection was achieved within three months after diagnosis in 113 of these patients (71.1 %). Thirty-four of the 159 patients (21.4 %) had incomplete resection and in 12 patients (7.5 %) biopsy only was performed. Hence, in 46 of 159 patients (28.9 %) residual tumor was observed for longer than three months. Twelve of these 46 patients had delayed surgery after 4 to 47 months, achieving complete resection only in four of those 12 patients. Forty-two patients with residual GN are currently under observation (median observation time: 42 months; range 1-110 months). Twenty-five of those 42 patients have major residuals (>2 cm), while in 14 only a minor residual was left. Information about the size of residual tumor was not available in three patients.

For 55 patients with GNBI data on extent of initial surgery was available. Tumor was completely resected within three months after diagnosis in 37 patients (67.3 %). Twelve patients (21.8 %) had incomplete resection and six patients (10.9 %) had biopsy only. Of those 18 patients with residual tumor, 10 underwent delayed surgery after 4 to 81 months, resulting in complete resection in five patients. Thus, in 13 patients a residual GNBI is currently under observation (median observation time: 53 months; range 6-134 months). Nine of those 13 patients have major residuals (>2 cm) and 4 have minor tumor residuals.

Table 2 provides more detailed information of the 22 patients with GN and GNBI who underwent delayed surgery.

**Table 2:** Clinical course of patients with delayed surgery (> 3 months after diagnosis) (n = 22)

Age at diagnosis	Tumor localization	Tumor volume	Extent of primary surgery	Histology	Chemotherapy	Time from diagnosis to delayed surgery	Extent of secondary surgery	Current status	Reason for delayed surgery
18 mo – 5 yrs	Pelvic	179 ml	Macroscopic residuals	GN mature	None	32 mo	Complete resection	CR	Suspected Progression
11 – 18 yrs	Pelvic	680 ml	Macroscopic residuals	GN maturing	None	8 mo	Macroscopic residuals	PR	Progression before delayed surgery
11 – 18 yrs	Abdominal	n.a.	Biopsy	GN maturing	None	4 mo	Complete resection	CR	Planned surgery
5 – 11 yrs	Thoracic	n.a.	Macroscopic residuals	GN maturing	None	4 mo	Macroscopic residual	PR	Planned surgery
5 – 11 yrs	Thoracic	168 ml	Macroscopic residuals	GN maturing	None	6 mo	Macroscopic residuals	LFU	First surgery not in Germany
5 – 11 yrs	Abdominal	20 ml	Biopsy	GN maturing	None	9 mo	Near -complete resection	CR	Suspected Progression
5 – 11 yrs	Abdominal	120 ml	Macroscopic residuals	GN maturing	2 cycles	4 mo	Macroscopic residual	VGPR	No response to chemotherapy
5 – 11 yrs	Adrenal	56 ml	Macroscopic residuals	GN maturing	None	5 mo	Macroscopic residuals	VGPR	Planned surgery
18 mo – 5 yrs	Abdominal	90 ml	Biopsy	GN maturing	None	47 mo	Macroscopic residuals	VGPR	Progression before delayed surgery
18 mo – 5 yrs	Abdominal	282 ml	Biopsy	GN maturing	None	9 mo	Complete resection	CR	Suspected Progression
18 mo – 5 yrs	Thoracic	16 ml	Biopsy	GN maturing	None	6 mo	Macroscopic residual	VGPR	Suspected Progression
0 – 18 mo	Thoracic	26 ml	Biopsy	GN maturing	None	21 mo	Macroscopic residuals	PR	Suspected Progression
18 – 21 yrs	Abdominal	72 ml	Macroscopic residuals	GNBI	4 cycles	11 mo	Biopsy	VGPR	No response to chemotherapy
5 – 11 yrs	Thoracic	11 ml	Biopsy	GNBI	None	4 mo	Near -complete resection	CR	No regression during observation
5 – 11 yrs	Adrenal	44 ml	Macroscopic residuals	GNBI	4 cycles	5 mo	Near -complete resection	CR	No response to chemotherapy
5 – 11 yrs	Thoracic	42 ml	Biopsy	GNBI	None	11 mo	Complete resection	CR	Suspected Progression
18 mo – 5 yrs	Adrenal	150 ml	Biopsy	GNBI	4 cycles	6 mo	Complete resection	CR	No response to chemotherapy
18 mo – 5 yrs	Cervical	87 ml	Biopsy	GNBI	4 cycles	5 mo	Macroscopic residual	VGPR	No response to chemotherapy
18 mo – 5 yrs	Thoracic	n.a.	Macroscopic residuals	GNBI	9 cycles (+MT +RA)	17 mo	Macroscopic residual	PR	Clinical progression 3 months after diagnosis/No response to chemotherapy
18 mo – 5 yrs	Abdominal	240 ml	Biopsy	GNBI	6 cycles (+MT +RA)	7 mo	Near -complete resection	CR	No response to chemotherapy
18 mo – 5 yrs	Thoracic	39 ml	Biopsy	GNBI	7 cycles	4 mo 10 mo 81 mo	Macroscopic residual / Macroscopic residual / Biopsy	PD	No response to chemotherapy/ Progression before surgery
18 mo – 5 yrs	Pelvic	98 ml	Macroscopic residuals	GNBI	None	22 mo	Macroscopic residual	VGPR	Suspected Progression

GNBI = ganglioneuroblastoma intermixed, GN = ganglioneuroma, n.a. = data not available, yrs = years, mo = months, MT = maintenance therapy, RA = retinoic acid,

CR = complete remission, VGPR = very good partial response, PR = partial response, SD = stable disease, PD = progressive disease, LFU = lost to follow-up

### Chemotherapy (Table 3)

**Table 3:** Clinical course of patients who received cytotoxic treatment (CT) (n = 11)

Age at diagnosis	Tumor localization	Tumor volume	Tumor stage	Extent of surgery prior to chemotherapy	Histology	CT	Response to CT	Current status	Remarks
11 – 18 yrs	Abdominal	768 ml	3	Biopsy	GN mature	2 x N5, 2 x N6, 5 x N7	SD	SD	Progression 18 months after end of maintenance therapy (N7)
5 – 11 yrs	Abdominal	120 ml	3	Macroscopic residuals	GN maturing	1 x N5, 1 x N6	SD	VGPR	Secondary surgery due to no response to chemotherapy
18 – 21 yrs	Abdominal	72 ml	2a	Macroscopic residuals	GNBI	2 x N5, 2 x N6	SD	VGPR	Secondary surgery due to no response to chemotherapy
5 – 11 yrs	Adrenal	44 ml	2a	Macroscopic residuals	GNBI	2 x N5, 2 x N6	SD	CR	Secondary surgery due to no response to chemotherapy
5 – 11 yrs	Pelvic	102 ml	2a	Macroscopic residuals	GNBI	3 x N4, 1 x N5	PR	LFU	LFU after 18 months
18 mo – 5 yrs	Adrenal	150 ml	3	Biopsy	GNBI	2 x N5, 2 x N6	SD	CR	Secondary surgery due to no response to chemotherapy
18 mo – 5 yrs	Cervical	87 ml	2a	Biopsy	GNBI	2 x N5, 2 x N6	SD	VGPR	Secondary surgery due to no response to chemotherapy
18 mo – 5 yrs	Thoracic	n.a.	3	Macroscopic residuals	GNBI	4 x N5, 4 x N6, 4 x N7, 1 x N8, 1 x RA	PR	PR	Progression of paraplegia 3 months after diagnosis
18 mo – 5 yrs	Abdominal	240 ml	3	Biopsy	GNBI	3 x N5, 3 x N6, 4 x N7, 9 x RA	PR	CR	Secondary surgery due to poor response to chemotherapy
18 mo – 5 yrs	Thoracic	39 ml	2a	Biopsy	GNBI	2 x N5, 2 x N6, 2 x N8, 1 x TE	SD	PD	Progression 71 months after last cycle of chemo therapy
0 – 18 mo	Adrenal	20 ml	1	Complete resection	GNBI	2 x N5, 2 x N6	-	Dead	Death of chemotherapy-related pulmonary edema

GNBI = ganglioneuroblastoma intermixed, GN = ganglioneuroma, n.a. = data not available, yrs = years, mo = months,

CR = complete remission, VGPR = very good partial response, PR = partial response, SD = stable disease, PD = progressive disease, LFU = lost to follow-up,

N5 = cisplatin/etopiside/vindesine, N6 = vincristine/dacarbacin/ifosfamide/doxorubicine, N7 = cyclophasphamide (oral), N8 = topotecan/cyclophasphamide/etoposide,

TE = topotecan/etoposide

Cytotoxic treatment was given to two patients with GN and 9 patients with GNBI. One patient with GN presented with a large stage 3 tumor and received chemotherapy because diagnosis of GN was made only from biopsy and immature components within the residuals were suspected by local physicians. The other patient with GN received chemotherapy because of an intraspinal tumor mass. No patient showed significant response to chemotherapy and residual tumor is still observed.

Nine patients with GNBI received cytotoxic treatment (stage 3 n = 3; stage 2a n = 5, stage 1 n = 1). Any response to treatment was only seen in three patients. In two of these patients, tumor size slightly decreased during the first two cycles of chemotherapy, while additional chemotherapy showed no effect. The third patient did not respond to frontline chemotherapy but subsequent to incomplete tumor resection, a reduction of tumor size was observed under chemotherapy. However, no patient achieved complete or very good partial response as substantial effect of chemotherapy. In six patients who have received chemotherapy residual tumor is still under observation.

### Outcome

**Fig. 3:**
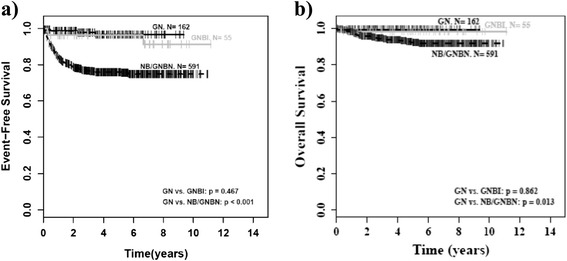
Outcome by histology. NB = neuroblastoma, GNBN = ganglioneuroblastoma nodular, GNBI = ganglioneuroblastoma intermixed, GN = ganglioneuroma. **a**) Event-free survival (EFS) by histology, **b**) Overall survival (OS) by histology

The survival curves in Fig. 3a and 3b present the excellent prognosis of GN and GNBI. Two patients died of treatment related complications. One patient with GN died of surgery related complications after complete tumor resection in initial surgery. One patient with GNBI died of heart failure due to chemotherapy related pulmonary edema. However, no patient died of tumor progression.

**Fig. 4:**
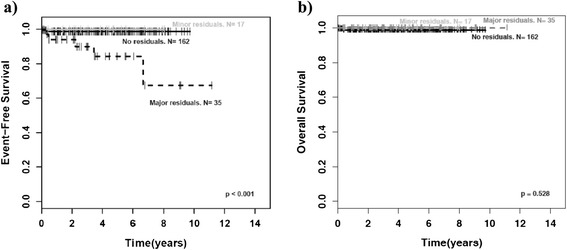
Outcome of GN/GNBI by tumor residuals. GNBI = ganglioneuroblastoma intermixed, GN = ganglioneuroma. **a**)EFS for GN/GNBI by tumor residuals, **b**) OS for GN/GNBI by tumor residuals, p = major residuals vs. no or minor residuals

**Fig. 5:**
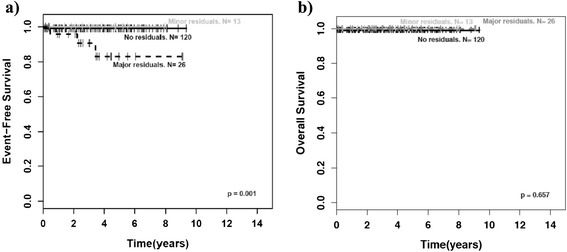
Outcome of ganglioneuroma (GN) by tumor residuals. **a**) EFS for GN by tumor residuals, **b**) OS for GN by tumor residuals, p = major residuals vs. no or minor residuals

**Fig. 6:**
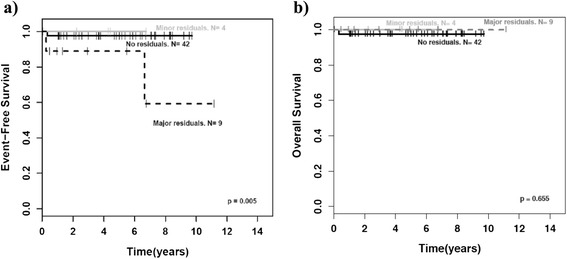
Outcome of ganglioneuroblastoma intermixed (GNBI) by tumor residuals. **a**) EFS for GNBI by tumor residuals, **b**) OS for GNBI by tumor residuals, p = major residuals vs. no or minor residuals

Event-free survival (EFS) of patients with incomplete tumor resection was not inferior to that of patients with complete resection if tumor residuals were smaller than 2 cm (minor residuals; p = 1.00). Of interest, this applied as well to the group of patients with GNBI taken by itself (p = 1.00). However, patients with major residuals (>2 cm) had a worse EFS than patients with complete tumor resection or minor residuals as well for the whole group of mature NT (p < 0.001; Fig. 4a) as for the groups of GN (p = 0.001; Fig. 5a) and GNBI seen individually (p = 0.005; Fig. 6a). Overall survival was not influenced by extent of initial surgery (Figs. 4b, 5b, and 6b).

Tumor progression was diagnosed in five patients (3 GN, 2 GNBI). Time to progression ranged from 3 to 80 months (median: 27 months). Four progressions were diagnosed via MRI, all of which were confirmed by central review. In three patients, progression of tumor size exceeded 25 % as required by INRC [21]. In the fourth patient, tumor progression was diagnosed due to newly developed intraspinal involvement. The fifth progression was diagnosed in a patient with GNBI by development of progressive paraplegia after 2 cycles of chemotherapy and incomplete resection. This patient immediately underwent incomplete resection of the intraspinal tumor without performing MRI or computed tomography before surgery.

Three of five patients with progressive disease had been treated with chemotherapy prior to progression (1 GN, 2 GNBI). No progression was seen in the subgroup of eight out of 13 patients with residual GNBI that did not receive cytotoxic treatment.

All five patients with progression had major tumor residuals (>2 cm). Three of the five tumors had been initially diagnosed from biopsies. The remaining two had incomplete initial resections, both with major tumor residuals. No progression was seen after complete resection or in patients with minor tumor residuals (<2 cm), neither in patients with GN nor in patients with GNBI.

After progression was diagnosed, three patients were treated with incomplete tumor resection and one underwent biopsy. The remaining patient was further observed after first progression and even showed second progression 21 months after first progression. However, thereafter tumor has been stable. All patients with progression are still under observation with residual tumor - four with major residuals and one with a minor tumor residual. Only one of these patients received chemotherapy after progression. Further information on all patients with tumor progression is shown in Tables 2 and 3.

## Discussion

This study analyzed one of the largest series of patients with GN/GNBI, demonstrating that they account for about one quarter of all localized neuroblastic tumors (NT). They show significant differences to immature NT (NB/GNBN) concerning age at diagnosis, stage, localization, tumor volume, mIBG-uptake, catecholamine metabolite excretion, NSE, and status of molecular markers (MYCN and 1p). Outcome was excellent and chemotherapy seems not effective while incomplete resection with minor residuals (<2 cm) appears to be sufficient for treatment.

In our study, the portion of GN was 20 % of all localized NT which is higher than in the majority of other published studies. In their large series of GN, De Bernardi et al found 14 % GN [18]. Generally, GN are likely to be under-represented in oncological trials due to the fact that benign tumors as GN are registered to the trial offices on a voluntary basis. We consider the higher percentage of GN observed in Germany as a reporting effect resulting from the close cooperation with the nationwide Deutsches Kinderkrebsregister (German Childhood Cancer’s Registry) over the last two decades. Alignment with their data reveals that nearly all patients in Germany with NT are registered in the German neuroblastoma trials [28].

The portion of GNBI (55 of 808 localized tumors; 6.8 %) appears low in our series. The ratio between GNBI (*n* = 55) and GN maturing (*n* = 144) was 1:2.6. This is much lower compared to the findings of Okamatsu et al and Cohn et al with ratios of 4.6:1 (198 GNBI, 43 GN maturing) and 3.7:1 (144 GNBI, 39 GN maturing) [19, 20]. Nonetheless, the ratio between GNBI and all immature NT (localized or metastatic; *n* = 1351) was 1:24.6. This is comparable to the findings of Okamatsu et al and Cohn et al where the ratio was 1:18.7 (198:3712) and 1:27 (144:3889) [19, 20]. Thus, the low portion of GNBI is artificial and results from the high completeness of included GN in our series.

Moreover, the border-line between GNBI and GN maturing - which was known as ganglioneuroblastoma borderline in the terminology proposed by Joshi et al [29] - might be set slightly different between individual pathologists. So, if central review is done by one pathologist a systematic shift to GN or GNBI might be possible.

It has been hypothesized that a good part of GN originates from neuroblastoma. This has first been suggested by Cushing in 1926 [5] and has since been suspected repeatedly for stage 4 s [11, 14, 30, 31] as well as for localized neuroblastoma [24, 32]. However, whether this is true for all GN, as proposed by Shimada [2], still remains unproven. An indirect support for this hypothesis can be seen by our observation that patients with GN and GNBI are older at diagnosis than those with immature NT. As Fig. 1 shows, the grade of neuroblastic differentiation increases with the median age at diagnosis. This is in line with the theory that mature NT might just have had enough time to mature in situ before they were discovered.

Besides, the widely spread medical checkups in infants and young toddlers in Germany with the use of ultrasound could cause a higher number of asymptomatic, small localized immature neuroblastomas found in infancy before they can grow larger and potentially cause symptoms. Here, further research is needed.

Outcome was excellent with no difference between GN and GNBI. Incomplete resection was not associated with increased risk of progression if tumor residuals were smaller than 2 cm. However, tumors that showed local progression had large tumor residuals after incomplete resection or biopsy. Therefore, it cannot be ruled out that the large tumor masses contained immature components as the sources of progression. In our study, no progression occurred after complete resection and subtotal resection with residuals < 2 cm in both, GN and GNBI. This supports the proposals of Duheme-Tonnelle et al [16] and Hayes et al [33] and others that resection does not have to be radical for the treatment of GN and GNBI. Instead subtotal resection without endangering vital structures seems to be sufficient. This is especially important as De Bernardi et al reported a high rate of surgical complications [18]. However, the extent of initial surgery should be sufficient to rule out immature areas within the tumor. Progressions only occurred in tumor residuals that were larger than 2 cm in diameter. However, as the overall survival of patients with progression was unimpaired after reoperation even larger tumor residuals might be acceptable to avoid risky surgery. Since progression often occurs subtle and long after initial diagnosis regular examinations of residual tumors are mandatory. Further, we found little to no benefit from cytotoxic treatment for patients with localized GNBI. Since only a very small number of patients with GNBI have received chemotherapy, it is possible that single patients may profit from cytotoxic treatment especially in case of immature regions within large tumor residuals. Our findings are in line with the results of De Bernardi et al who also demonstrated an excellent outcome for patients with GNBI without cytotoxic treatment [18]. Thus, chemotherapy seems not to be indicated for patients with localized GNBI similar as for patients with GN.

To our knowledge, this is the first analysis reporting on the influence of the size of residual tumor on EFS and the poor effect of chemotherapy in mature neuroblastic tumors.

An interesting question is why mature tumors can show progression at all. Nishihira et al suggested that a minor increase of tumor volume may also be caused by proliferation of Schwannian stroma rather than that of tumor cells [34]. This may explain limited variations in tumor volume not reaching 25 % as required for diagnosis of tumor progression according to INRC [21].

## Conclusions

In conclusion, clinical features and behavior are very similar for GN and GNBI and outcome is excellent for both. Cytotoxic treatment has no substantial effect on both tumor entities. Surgery alone is sufficient for the treatment of GN and GNBI and does not need to be radical if only minor residuals are left (e.g. < 2 cm). For patients diagnosed with GN or GNBI, subtotal, non-mutilating resection and regular long-term follow up are warranted.

## Abbreviations

1p, Short arm of chromosome 1; EFS, Event-free survival; GN, Ganglioneuroma; GNBI, Ganglioneuroblastoma intermixed; GNBN, Ganglioneuroblastoma nodular; GPOH, German Association for Pediatric Oncology and Hematology (Gesellschaft für pädiatrische Onkologie und Hämatologie); INPC, International Neuroblastoma Pathology Classification; INRC, International Neuroblastoma Response Criteria; INSS, International Neuroblastoma Staging System; mIBG, Metaiodobenzylguanidine; MRI, Magnetic resonance imaging; MYCN, Oncogene MYCN; NB, Neuroblastoma; NSE, Neuron specific enolase; NT, Neuroblastic tumors; OS, Overall survival

## Additional file

Additional file 1: Dataset (PDF 291 kb)
