# Supplementary material for: Treatment and outcome of Ganglioneuroma and Ganglioneuroblastoma intermixed
Source: BMC Cancer. 2016 Jul 27;16:542. doi: 10.1186/s12885-016-2513-9 (PMC4964292; doi:10.1186/s12885-016-2513-9)
Supplement: Additional file 1: — Dataset (PDF 291 kb) [file 12885_2016_2513_MOESM1_ESM.pdf]

Gruppe	PAT_ALTER	STADIUM	REMDAU	UEBDAU	STATUS	EVENT_ART	LOH	NMYC	MinvonTUMO	MaxvonINTRA	TU_HOCH
GN mature	3697	3	604	604	0			1	4	1	4,9
GN mature	3591	1	374	374	0				3		4
GN mature	3955	1	80	80	0		-1	-1	3		5,9
GN mature	3895	1	310	310	0				4		8
GN mature	4626	2,1	153	153	0		2	1	4		13,5
GN mature	1156	0	53	53	0		2	1	0		
GN mature	2724	3	2874	2874	0			1	4		4
GN mature	5133	3	331	331	0				6	1	10
GN mature	4603	3	813	2495	2	4		1	3		8
GN mature	5154	1	522	522	0			1	3		6
GN mature	4835	1	887	887	0				1		3,5
GN mature	2830	1	1450	1450	0				3		3
GN mature	3522	1	1839	1839	0				1		7,8
GN mature	1675	1	1620	1620	0				4		
GN mature	1482	1	1161	1161	0				3		8,4
GN mature	840	3	1583	1583	0			1	6		7
GN mature	3985	1	734	734	0		-1	-1	1		11,6
GN mature	5260	2,1	1117	1117	0		2	1	3		9,9
GN maturing	2124	3	552	552	0		2	1	1		11,7
GN maturing	900	1	73	73	0			1	1		3
GN maturing	2124	1	453	453	0				6		6,3
GN maturing	1944	3	339	339	0		2	1	3	1	5
GN maturing	1420	1	384	384	0		2	1	6		4,5
GN maturing	1659	-3	81	81	0		-1	-1	3		3,8
GN maturing	4102	1	67	67	0			1	1		6,4
GN maturing	1406	1	442	442	0			1	1		
GN maturing	2815	2,1	131	131	0				4		3,5
GN maturing	5704	2,1	385	385	0				3		3,5
GN maturing	1951	1	153	153	0			1	4		2
GN maturing	3395	2,1	1619	1619	0		2	1	4		10
GN maturing	1669	1	1806	1806	0		2	1	1		4,5
GN maturing	2922	1	425	425	0			1	1		4,7
GN maturing	3176	2,1	2886	2886	0			1	1		5
GN maturing	1328	1	2409	2409	0		2	1	3		6,2
GN maturing	3961	3	3331	3331	0		2	1	4		
GN maturing	1500	1	2352	2352	0				3		3,8
GN maturing	3042	1	26	26	0				1		2,1
GN maturing	3438	1	105	105	0		2	1	4		5,9
GN maturing	2064	1	1913	1913	0				4		14
GN maturing	1389	1	1997	1997	0			1	1		6
GN maturing	4108	2,1	2211	2211	0			1	4		3,5
GN maturing	1317	1	2435	2435	0				5		3,2

GN maturing	5918	1	800	800	0				1		
GN maturing	2121	1	3413	3413	0				3		4,6
GN maturing	1522	1	3219	3219	0		2	1	3		3
GN maturing	3408	1	3415	3415	0				3		3,1
GN maturing	4009	1	2708	2708	0				3		10
GN maturing	3114	1	994	994	0				1		3,8
GN maturing	4362	1	1580	1580	0			1	3		8
GN maturing	4095	1	2118	2118	0			1	3		8
GN maturing	1934	1	1995	1995	0			1	6		
GN maturing	1827	2,1	54	54	0		2	1	4		4,9
GN maturing	1925	1	2811	2811	0			-1	3		8
GN maturing	3507	1	1988	1988	0				3		4
GN maturing	2008	1	2871	2871	0			1	5		4,8
GN maturing	4847	1	2295	2295	0			1	1		
GN maturing	2062	1	1845	1845	0			1	1		10,8
GN maturing	3064	-1	1361	1361	0	0		1	4	1	9,5
GN maturing	1596	1	1938	1938	0			1	1		3,6
GN maturing	975	1	2478	2478	0			1	1		1,5
GN maturing	3652	1	321	321	0				6		11
GN maturing	1082	2,2	2733	2733	0			1	4		
GN maturing	1638	1	146	146	0			1	4		
GN maturing	4627	1	2960	2960	0	0	-1	1	3		6,3
GN maturing	1360	1	2322	2322	0		-1		4		2,2
GN maturing	3355	2,1	776	776	0			1	4	1	9,5
GN maturing	2319	1	2947	2947	0				1		5
GN maturing	3181	-2	937	937	0			1	4		8
GN maturing	3518	1	848	848	0				1		6,3
GN maturing	4494	1	2386	2386	0			1	1		3,6
GN maturing	1296	1	281	281	0		2	1	1		1,6
GN maturing	3027	1	2259	2259	0		-1		1		8
GN maturing	1372	0	7	7	0				6	1	
GN maturing	956	1	2180	2180	0			1	3		2,5
GN maturing	3551	1	1799	1799	0			1	3		1,8
GN maturing	5103	1	1577	1577	0				1		6
GN maturing	4803	1	2179	2179	0			1	1		6
GN maturing	1567	1	248	248	0			1	3		3
GN maturing	2659	1	2296	2296	0				1		3,5
GN maturing	1354	1	1150	1150	0	0		1	1		3
GN maturing	2106	-1	1937	1937	0	0	2	1	4		6,9
GN maturing	3805	1	1878	1878	0				1		9,5
GN maturing	3015	1	1134	1134	0	0			3		7,2
GN maturing	2693	1	2051	2051	0		2	1	4		4,5
GN maturing	1840	1	2541	2541	0				3		5,1

GN maturing	4640	2,1	1344	1344	0				1		7
GN maturing	1921	2	2023	2023	0				1	1	3,6
GN maturing	590	1	1238	1238	0		2	1	5		2,2
GN maturing	2864	1	2387	2387	0			1	3		8
GN maturing	1294	1	2620	2620	0		2	1	1		3
GN maturing	2087	1	2934	2934	0	0	-1	-1	5		3
GN maturing	2798	2,1	2096	2096	0			1	3		4,1
GN maturing	1201	1	1532	1532	0			1	3		4,8
GN maturing	3747	1	2401	2401	0		-1	-1	3		4
GN maturing	5398	1	456	456	0				1		5
GN maturing	2002	1	2012	2012	0	0	2	1	1		2,6
GN maturing	3362	3	1912	1912	0			1	4	1	
GN maturing	1801	3	1717	1717	0			1	3	1	
GN maturing	3435	1	930	930	0			1	3		3,9
GN maturing	4881	1	1587	1587	0			1	1		5
GN maturing	2815	1	2106	2106	0				3		4
GN maturing	1039	2,1	2154	2154	0				5	1	2,4
GN maturing	3922	1	805	805	0				4		3
GN maturing	1342	1	532	532	0			1	4		6
GN maturing	2203	1	2085	2085	0			1	3		3,3
GN maturing	5605	1	804	804	0			1	1		6
GN maturing	4766	1	891	891	0				1		9
GN maturing	1695	-2,2	2022	2022	0			1	1		8
GN maturing	1523	1	24	24	1				1		10
GN maturing	2434	2,1	1812	1812	0				4		
GN maturing	5159	1	1741	1741	0			-1	4		5
GN maturing	2790	1	1445	1445	0				3		4
GN maturing	6074	1	692	692	0				1		5,3
GN maturing	1858	1	104	104	0		2	1	3		2,5
GN maturing	1580	2,1	1737	1737	0			1	4	1	
GN maturing	2772	1	1726	1726	0				4		10
GN maturing	1344	-2,1	35	35	0				3		
GN maturing	3367	1	1270	1270	0			1	1		
GN maturing	4824	3	1819	1819	0			-1	3	1	9,5
GN maturing	3408	1	1354	1354	0			1	4		7
GN maturing	1612	1	1548	1548	0		2	1	3		8
GN maturing	1775	2,1	1634	1634	0				4		
GN maturing	1468	3	1435	1435	0		2	1	3		9,4
GN maturing	6498	1	1525	1525	0				1		4
GN maturing	1535	2,1	1253	1472	2	4			3		7,5
GN maturing	2024	3	1113	1113	0				6		3
GN maturing	681	1	1678	1678	0				1		5,6
GN maturing	1050	1	1451	1451	0				3		6

GN maturing	1349	1	1132	1132	0				6		1,8
GN maturing	4844	1	299	299	0				1		3,1
GN maturing	4667	2,1	1424	1424	0				3		
GN maturing	2840	2,1	1532	1532	0				3		4
GN maturing	1483	1	1437	1437	0		2	1	1		3,8
GN maturing	1215	3	1417	1417	0		2	1	3		4
GN maturing	2789	1	902	902	0		-1	1	3		7,5
GN maturing	5287	2	1299	1299	0	0			1		
GN maturing	2083	1	1193	1193	0		2	1	1		2,5
GN maturing	1494	1	1067	1067	0		2	1	3		4
GN maturing	3053	2,1	1314	1314	0	0	2	1	1		9
GN maturing	2193	1	389	389	0				6		24,4
GN maturing	2081	1	643	643	0			1	4		5
GN maturing	4453	1	929	929	0				3		4
GN maturing	1446	1	1237	1237	0				5		2
GN maturing	1417	2,1	1092	1092	0		2	1	4		3,5
GN maturing	1614	1	683	683	0		2	1	1		
GN maturing	2888	1	947	947	0			1	3	1	4,1
GN maturing	309	3	1096	1096	0		-1	1	4	1	1,9
GN maturing	1779	1	579	579	0			1	1		10
GN maturing	2954	1	901	901	0				3		
GN maturing	1333	1	677	677	0		-1	1	4		10
GN maturing	4860	2,1	798	798	0				6	1	8
GN maturing	4004	1	894	894	0			1	3		9
GN maturing	1097	3	950	950	0		-1	1	4		2
GN maturing	2083	3	763	763	0	0		1	3	0	
GN maturing	1982	1	771	771	0			1	1		8,5
GN maturing	2960	2,1	899	899	0				4		4,2
GN maturing	2613	1	420	420	0		-1	1	1		7
GN maturing	2723	2,1	671	671	0		2	1	3		3
GN maturing	1109	1	513	513	0		2	1	6	1	6,3
GN maturing	775	1	785	785	0		-1	1	3		4,9
GN maturing	1146	1	75	75	0				3		2,3
GN maturing	3243	2,1	853	853	0				4		2,3
GNBI	1319	1	586	586	0		-1	1	4		2,2
GNBI	768	1	396	396	0		2	1	4		1
GNBI	2895	1	444	444	0	0		1	1		4
GNBI	2931	2,1	483	483	0		2	1	4	1	8,6
GNBI	974	-2,2	409	409	0		2	1	4		2,6
GNBI	2121	1	308	308	0			1	3		11
GNBI	1638	2,1	4073	4073	0			1	3		4,3
GNBI	958	1	2587	2587	0		2	1	1		3,2
GNBI	1065	1	3549	3549	0			1	1		2,3

GNBI	521	1	119	119	1		2	1	1		3,2
GNBI	4669	1	2083	2083	0		-1	1	3		
GNBI	2201	1	3356	3356	0		1,1	1	4		4,5
GNBI	1319	1	941	941	0			1	1		4
GNBI	1203	1	3485	3485	0			1	3	1	3,8
GNBI	1917	1	2146	2146	0			1	1		5,7
GNBI	7640	2,1	2470	2470	0		2	1	3	1	6
GNBI	1964	1	2457	2457	0			1	6		4,4
GNBI	936	1	1384	1384	0		2	1	4		7,5
GNBI	1602	2,1	1564	1564	0			1	4		
GNBI	2477	1	3034	3034	0			1	1		8,2
GNBI	2364	1	3085	3085	0			1	3		
GNBI	589	2,1	2893	2893	0			1	5		2,5
GNBI	3058	1	12	12	0			1	4		
GNBI	2022	2,1	3046	3046	0		2	1	4	1	4
GNBI	1265	2,1	818	818	0			1	5		
GNBI	455	1	3016	3016	0		-1	1	6		3
GNBI	1894	2,1	174	174	0			1	6		4
GNBI	1310	1	2689	2689	0		-1	1	3		15,5
GNBI	2036	2,1	2668	2668	0		2	1	1		4,4
GNBI	1824	1	2891	2891	0				1		5,5
GNBI	3182	1	2665	2665	0			1	4		
GNBI	2249	1	1886	1886	0		2	1	1		6
GNBI	2931	2,1	2461	2461	0			1	3	1	
GNBI	3788	1	2369	2369	0			1	3		
GNBI	2577	1	2227	2227	0		2	1	4		3
GNBI	957	2,1	2424	2468	2	4	2	1	4		3,8
GNBI	1860	1	2325	2325	0			1	1		3,3
GNBI	3128	1	2219	2219	0		-1	-1	5		2
GNBI	1007	1	378	378	0	0	2	1	1		2,5
GNBI	700	1	1707	1707	0	0	2	1	1		2
GNBI	3465	1	1963	1963	0	0	2	1	3		8
GNBI	1976	1	1757	1757	0		2	1	3		
GNBI	1191	3	1829	1829	0			1	3		8
GNBI	1513	2,1	1613	1613	0		2	1	3		3,5
GNBI	549	-2,1	1808	1808	0		2	1	6	1	4,3
GNBI	3776	1	1093	1093	0			1	4		3,6
GNBI	1285	3	95	1796	2	4	2	1	4	1	
GNBI	887	2,1	1073	1073	0	0	-1	1	3	1	7
GNBI	3570	2,1	1333	1333	0		2	1	4		4,6
GNBI	2973	1	761	761	0		2	1	3		3,7
GNBI	3699	1	989	989	0			1	3		7
GNBI	1491	3	349	349	0		-1	1	1	1	13,1

GNBI	1309	1	873	873	0		-1	1	4		6
GNBI	2632	1	753	753	0	0	2	1	3		
GNBI	1271	3	655	655	0		2	1	1		5
NB/GNBN	123	2,1	799	799	0		2	1	1		2,5
NB/GNBN	451	2,2	697	697	0		2	1	4		4
NB/GNBN	657	1	687	687	0		2	1	1		2,5
NB/GNBN	531	1	340	340	0		2	1	6		1,4
NB/GNBN	191	3	569	569	0		2	1	3	1	3,4
NB/GNBN	323	3	780	780	0		2	1	5		
NB/GNBN	1507	3	511	769	2	4	1,1	1	4	1	2,5
NB/GNBN	762	1	311	311	0		2	1	1		1,8
NB/GNBN	451	2,1	726	726	0		2	1	4		5
NB/GNBN	0	1	86	86	0		2	1	4		3
NB/GNBN	257	2	112	763	2	4	2	1	5		4,5
NB/GNBN	34	2,1	41	726	2	4	2	1	1	1	
NB/GNBN	216	1	316	316	0		1,1	1	6		4,6
NB/GNBN	292	2,1	690	690	0		2	1	4		3,2
NB/GNBN	317	1	610	610	0		1,1	1	3		5,1
NB/GNBN	173	3	458	929	1	4	2	1	3		
NB/GNBN	0	-2,1	199	199	0	0	2	1	1		2
NB/GNBN	396	2,2	409	409	0		2	1	3		4,3
NB/GNBN	133	2,2	264	264	0		2	1	3		3,1
NB/GNBN	61	1	450	450	0		2	1	1		2,2
NB/GNBN	383	3	417	417	0	0	2	1	6	1	
NB/GNBN	87	2,1	280	676	2	2	2	1	4	1	2
NB/GNBN	58	2,1	628	628	0		2	1	6		4,7
NB/GNBN	1817	2,2	614	614	0		2	1	3		4,5
NB/GNBN	0	2,1	125	674	2	4	2	1	3		4,5
NB/GNBN	493	2,2	638	638	0		2	1	3		5,2
NB/GNBN	345	2,1	573	573	0		2	1	6	1	1,8
NB/GNBN	0	2,1	561	561	0		2	1	5		3
NB/GNBN	1117	1	221	221	0		2	1	1		
NB/GNBN	231	3	550	550	0		2	1,5	3	1	7,2
NB/GNBN	210	1	477	477	0		4	15	1		2,5
NB/GNBN	713	3	258	258	0		-1	100	1		
NB/GNBN	555	2,1	326	326	0		2	1	4		3
NB/GNBN	564	1	246	246	0		2	1	3		3,2
NB/GNBN	429	3	376	376	0		2	1	3	1	
NB/GNBN	21	3	297	583	2	4	1,1	1	6		7,1
NB/GNBN	1070	3	343	343	0		2	1,5	3		10,8
NB/GNBN	36	2,1	194	194	0		2	1	4	1	6
NB/GNBN	70	2,2	540	540	0		2	1	5		1,2
NB/GNBN	169	2,1	266	602	2	4	2	1	4		0,9

NB/GNBN	47	2,1	34	34	0		2	1	3	1	2,4
NB/GNBN	594	1	15	15	0		2	1	1		3,7
NB/GNBN	598	2,2	350	350	0		2	1	1		
NB/GNBN	1272	3	382	387	2	4	4	50	3		6,9
NB/GNBN	394	3	169	538	2	4	2	1	3		5,5
NB/GNBN	373	3	275	520	2	4	2	1	3		4,4
NB/GNBN	0	1	239	483	2	2	2	1	1		
NB/GNBN	0	1	189	189	0		2	1	1		1,8
NB/GNBN	65	3	357	547	2	4	-1	-1	1		
NB/GNBN	1676	1	3814	3814	0			1	4	1	2,1
NB/GNBN	371	1	3583	3583	0		1,1	1	1		
NB/GNBN	374	2,1	134	3385	2	4	2	1	3	1	5,4
NB/GNBN	453	2,1	651	2773	2	4	2	1	3	1	4,1
NB/GNBN	434	1	3993	3993	0		2	1	4		2,5
NB/GNBN	365	3	3983	3983	0		2	1	3		
NB/GNBN	176	2,1	89	3896	2	4		1	3	1	5
NB/GNBN	272	1	2549	2549	0		2	1	4		2
NB/GNBN	163	1	2467	2467	0		2	1	1		4
NB/GNBN	456	1	1961	1961	0		2	1	3		7
NB/GNBN	3	1	79	3638	2	2	2	1	1		5,5
NB/GNBN	740	1	2905	2905	0		1	1	3		4
NB/GNBN	452	2,2	2619	2619	0		2	1	1		3,5
NB/GNBN	1232	3	602	3620	2	2	2	1	3		4,9
NB/GNBN	276	2,1	2747	2747	0		2	1	4		1,7
NB/GNBN	425	1	2355	2355	0		2	1	1		1,5
NB/GNBN	565	1	1202	1202	1		2	1	1		5
NB/GNBN	245	1	3209	3209	0			1	1		4
NB/GNBN	245	1	2933	2933	0		2	1	3		5
NB/GNBN	428	2,1	3836	3836	0		4	10	4		2
NB/GNBN	66	2,1	3185	3185	0		2	1	3	1	4,1
NB/GNBN	207	2,2	674	1820	1	4	2	1	1		4,5
NB/GNBN	465	1	3634	3634	0		4	1	1		5,5
NB/GNBN	429	2,1	3411	3411	0		2	1	3		6
NB/GNBN	449	1	3352	3352	0		2	1	1		4,2
NB/GNBN	216	1	2184	2184	0		4	1	1		4,2
NB/GNBN	12	3	52	2255	2	4	-1	1	3		5
NB/GNBN	83	1	3462	3462	0		2	1	1		
NB/GNBN	485	1	3805	3805	0		2	1	1		2
NB/GNBN	566	3	2366	2366	0		2	1	6	1	5
NB/GNBN	666	2,1	56	522	1	4	1,1	1	4	1	6
NB/GNBN	460	1	3080	3080	0		2	1	3		3,6
NB/GNBN	447	3	3720	3720	0			1	4	1	1,5
NB/GNBN	21	2,2	3640	3640	0		1	1	3		1,5

NB/GNBN	374	2,1	3261	3261	0		1	1	3	1	3,6
NB/GNBN	49	2,1	3339	3339	0		2	1	1		4,2
NB/GNBN	16	1	3540	3540	0		2	1	1		4,3
NB/GNBN	121	1	1772	1772	0		2	1	3		3,8
NB/GNBN	426	3	3405	3405	0		2	1	4	1	
NB/GNBN	0	1	2220	2220	0		2	1	6		3,8
NB/GNBN	117	1	3409	3409	0		1	1	4		4,5
NB/GNBN	82	1	3584	3584	0		2	1	1		3,7
NB/GNBN	47	3	3226	3226	0		2	1	3		3,8
NB/GNBN	497	1	3468	3468	0		2	1	1		3,2
NB/GNBN	114	2,1	72	3837	2	4	2	1	1		8,5
NB/GNBN	385	1	107	3283	2	2	2	1	4	1	2,9
NB/GNBN	10	1	2055	2055	0		2	1	1		4,5
NB/GNBN	123	1	3149	3149	0		2	1	3		7
NB/GNBN	155	1	1723	1723	0		2	1	1		2,2
NB/GNBN	367	3	2016	2016	0		2	1	1		6
NB/GNBN	58	1	3199	3199	0			1	1		
NB/GNBN	0	2,1	76	3747	2	4	2	1	1		
NB/GNBN	76	1	3437	3437	0			1	1		4,9
NB/GNBN	427	1	883	883	0		2	1	1		3
NB/GNBN	25	3	126	268	1	2		1	3	1	1,7
NB/GNBN	743	1	2226	2226	0		2	1	1		3
NB/GNBN	482	2,2	32	32	1		4	100	1		
NB/GNBN	156	2,1	78	3341	2	4	4	1	4		2,9
NB/GNBN	324	3	75	75	1		2	1	3	1	6,5
NB/GNBN	465	1	2673	2673	0	0	2	1	4		3,4
NB/GNBN	11	1	2434	2434	0			1	1		
NB/GNBN	1536	3	117	147	1	4	4	50	4	1	
NB/GNBN	225	1	2886	2886	0		2	1	1		
NB/GNBN	1000	3	402	416	1	4	4	64	3		
NB/GNBN	460	2,2	1748	1748	0		2	1	1		2,2
NB/GNBN	1690	1	3043	3043	0			1	1		6,5
NB/GNBN	460	2,1	2454	2454	0		2	1	3		2,7
NB/GNBN	480	2,1	3574	3574	0		2	1	4	1	3,3
NB/GNBN	1215	2,2	3236	3236	0		2	1	4	1	7
NB/GNBN	262	1	33	3759	2	2	2	1	6		3,5
NB/GNBN	469	3	3331	3331	0		2	1	1		2
NB/GNBN	1088	3	3756	3756	0			8	3		12
NB/GNBN	291	3	3236	3236	0		2	1	4		
NB/GNBN	468	1	1484	1484	0		2	1	1		5,5
NB/GNBN	381	2,2	3428	3428	0		2	1	1		4
NB/GNBN	317	2,2	1894	1894	0		2	1	5		3,2
NB/GNBN	0	1	2167	2167	0		2	1	1		4

NB/GNBN	1652	3	3526	3526	0		4	64	1		8
NB/GNBN	54	1	2740	2740	0			1	1		3
NB/GNBN	1	2	3293	3293	0		2	1	3	1	3
NB/GNBN	867	2,1	3452	3452	0		2	1	1		4,5
NB/GNBN	638	2,2	3290	3290	0		2	1	4		2,5
NB/GNBN	91	2,2	3208	3208	0		2	1	1		6,2
NB/GNBN	507	2,2	3508	3508	0		2	1	3		7
NB/GNBN	2831	3	685	3468	2	2	2	1	1		7,2
NB/GNBN	30	1	2548	2548	0		2	1	3		
NB/GNBN	151	3	2088	2088	0			1	4	1	
NB/GNBN	470	3	2594	2594	0		2	1	1		
NB/GNBN	887	1	3487	3487	0		2	1	1		5
NB/GNBN	94	1	2504	2504	0	0	2	1	3		6
NB/GNBN	1628	3	352	423	1	4		16	3		9,5
NB/GNBN	2440	1	1845	1845	0			1	1		10
NB/GNBN	123	1	430	430	0		2	1	3		
NB/GNBN	340	2,2	3467	3467	0		2	1	3		3,8
NB/GNBN	31	2	86	3020	2	4		1	1		4,5
NB/GNBN	140	2,2	362	598	1	2		32	1		4
NB/GNBN	19	1	1647	1647	0			1	1		4,3
NB/GNBN	0	1	2706	2706	0		-1	1	1		5,3
NB/GNBN	222	2,1	3412	3412	0			1	4		7
NB/GNBN	528	2,1	2591	2591	0		2	1	4		3
NB/GNBN	971	2,1	3231	3231	0		2	1	4		1
NB/GNBN	473	2,2	2868	2868	0		2	1	4		
NB/GNBN	464	1	2928	2928	0		2	1	4		2,5
NB/GNBN	2169	2,2	277	3548	2	2	1,1	1	3		7
NB/GNBN	18	1	2175	2175	0		2	1	1		3
NB/GNBN	544	1	2432	2432	0		2	1	4		
NB/GNBN	3	3	181	1037	2	4	4	1	5		5
NB/GNBN	153	2,2	2648	2648	0			1	5		4
NB/GNBN	606	2,1	118	3336	2	4		1	4		4
NB/GNBN	406	1	3206	3206	0			1	1		2,5
NB/GNBN	190	2,1	2792	2792	0		2	1	1		
NB/GNBN	183	2,1	1742	1742	0		2	1	5		3
NB/GNBN	32	1	2422	2422	0	0		1	6		4,9
NB/GNBN	26	1	2992	2992	0			1	1		2,8
NB/GNBN	404	3	3047	3047	0			1	3		5,8
NB/GNBN	1643	3	3156	3156	0			1	3		10
NB/GNBN	218	1	3013	3013	0		2	1	1		4
NB/GNBN	31	1	0	0	1		2	1	1		3
NB/GNBN	1438	3	872	3307	2	4	2	1	3		5
NB/GNBN	105	2,1	2061	2061	0			1	4		3,5

NB/GNBN	199	-1	141	3092	2	4	2	1	4			4,1
NB/GNBN	206	2,1	132	3217	2	4	2	1	3			5,8
NB/GNBN	459	3	416	3234	2	4	2	1		3		7
NB/GNBN	204	1	2263	2263	0		2	1	1			9
NB/GNBN	349	2,1	2057	2057	0		2	1	3			6,5
NB/GNBN	864	3	2062	3303	2	2	4	20	1			
NB/GNBN	1495	3	1915	1992	5	5	1,1	1	1			10
NB/GNBN	208	2,1	3276	3276	0		2	1	4		1	5,5
NB/GNBN	1126	3	1246	3163	2	2	2	1	1			8,5
NB/GNBN	119	3	108	2621	2	4	2	1	3			4
NB/GNBN	39	1	85	3408	2	2		1	1			
NB/GNBN	502	1	1406	1406	0		2	1	4			5
NB/GNBN	344	3	3097	3097	0		2	1	4		1	
NB/GNBN	14	3	2086	2086	0			1	3			4
NB/GNBN	34	1	3326	3326	0		2	1	1			4,5
NB/GNBN	404	1	1703	1703	0		2	1	3			9
NB/GNBN	304	3	2988	2988	0		2	1	4		1	4,6
NB/GNBN	564	1	3149	3149	0	0	2	1	4			
NB/GNBN	727	3	371	3114	2	4	2	1	4		1	6
NB/GNBN	379	3	3304	3304	0		2	1	3			4,5
NB/GNBN	151	2,2	3324	3324	0		2	1	6			5
NB/GNBN	197	2,2	289	3614	2	4	2	1	1			2,5
NB/GNBN	63	1	2685	2685	0		2	1	1			5
NB/GNBN	47	3	1128	3330	2	4	2	1	3		1	7
NB/GNBN	33	1	1998	1998	0		2	1	1			2,5
NB/GNBN	0	2,1	208	2558	2	4	2	1	3		1	3,5
NB/GNBN	163	1	2304	2304	0			1	1			4,5
NB/GNBN	4562	2,2	759	1773	1	2	2	1	1			
NB/GNBN	37	3	119	2631	2	4	2	1	4		1	6
NB/GNBN	534	1	597	2913	2	2	-1	1	3			3,6
NB/GNBN	179	3	1771	1771	0		2	1	4		1	4,6
NB/GNBN	479	1	3115	3115	0		2	1	3			2,5
NB/GNBN	10	2,1	3105	3105	0			1	4		1	4,8
NB/GNBN	1457	1	1857	1857	0		2	1	3			5,4
NB/GNBN	9	2	2752	2752	0		2	1	4		1	3,4
NB/GNBN	292	1	1860	1860	0			1	5			
NB/GNBN	77	1	3122	3122	0		2	1	5			3
NB/GNBN	823	3	2978	2978	0		4	32	1			9
NB/GNBN	603	3	2151	2151	0			1	3			8
NB/GNBN	223	2	380	2627	2	4	2	1	3			5,8
NB/GNBN	114	2,1	2893	2893	0		2	1	4			3,5
NB/GNBN	0	1	443	443	0		2	1	3			5
NB/GNBN	270	2,2	29	3002	2	4	2	1	5			2,44

NB/GNBN	1639	3	200	755	2	4	2	1	5			2,5
NB/GNBN	366	1	2453	2453	0			1	1			1,2
NB/GNBN	6	2,1	192	3021	2	4	2	1	3		1	
NB/GNBN	515	2,2	2591	2591	0		2	1	1			5
NB/GNBN	4273	2,1	699	2741	2	2	1,1	1	1			12
NB/GNBN	26	2,2	2901	2901	0		2	1	1			2,84
NB/GNBN	7129	3	328	1969	1	4	2	1	3			3,5
NB/GNBN	810	2,2	916	1376	1	2	4	30	1			6
NB/GNBN	816	1	615	3010	2	2	4	1		3	1	3
NB/GNBN	303	1	2499	2499	0		2	1	6			6
NB/GNBN	1742	-3	2898	2898	0		4	64	1			6
NB/GNBN	149	1	1518	1518	0		2	1	3			5
NB/GNBN	734	1	2832	2832	0		2	10	1			5
NB/GNBN	470	3	3032	3032	0		4	100	1			
NB/GNBN	656	1	2893	2893	0			1	1			
NB/GNBN	314	1	2331	2331	0			1	4			
NB/GNBN	542	1	1946	1946	0			1	5			2,4
NB/GNBN	19	2,1	62	2561	2	4	2	1	3		1	1
NB/GNBN	0	1	2518	2518	0			1	1			3
NB/GNBN	5715	3	1878	2476	2	4	2	1	3		1	
NB/GNBN	112	2,1	2878	2878	0		2	1	4			5,1
NB/GNBN	10	1	2535	2535	0			1	5			
NB/GNBN	1465	1	1409	1409	0		2	1	3			3,5
NB/GNBN	228	2,2	2753	2753	0		2	1	1			5,4
NB/GNBN	848	3	2495	2495	0		4	50	3			15
NB/GNBN	4035	3	432	877	1	2	2	1	1			8
NB/GNBN	662	3	11	17	1	4	2	1	4		1	
NB/GNBN	49	1	2431	2431	0		2	1	1			
NB/GNBN	243	1	2828	2828	0		2	1	3			2,5
NB/GNBN	350	1	2576	2576	0		1,1	1	6			5
NB/GNBN	1381	3	2719	2719	0		2	1	4		1	
NB/GNBN	2737	3	2828	2828	0		1,1	64	1			7,2
NB/GNBN	248	1	3007	3007	0		1,1	1	1			2,8
NB/GNBN	203	2,1	2870	2870	0		2	1	1			2,3
NB/GNBN	0	1	2804	2804	0		2	1	3			2,7
NB/GNBN	1308	3	2787	2787	0		4	50	3			4,3
NB/GNBN	222	3	2552	2552	0		1,1	1	1			7,9
NB/GNBN	123	1	302	2807	2	2		1	3			3,8
NB/GNBN	230	2,1	2316	2316	0		1	1	1			4,3
NB/GNBN	7	2,2	2310	2310	0		2	1	3			7
NB/GNBN	61	2,1	1655	1655	0		2	1	3			2,6
NB/GNBN	410	1	2743	2743	0		2	1	1			1,6
NB/GNBN	335	2,2	2046	2046	0		4	32	1			

NB/GNBN	254	1	436	2936	5	5	2	1	3		1,8
NB/GNBN	4	1	2759	2759	0		2	1	4		5,9
NB/GNBN	240	2,1	54	2849	2	4	2	1	1		4,5
NB/GNBN	155	1	2009	2009	0		2	1	3		3
NB/GNBN	217	2,2	2414	2414	0		1,1	1	1		
NB/GNBN	1028	2	438	849	1	4	4	999	1	1	
NB/GNBN	220	1	2210	2210	0			1	1		5
NB/GNBN	35	1	2812	2812	0		2	1	1		3
NB/GNBN	38	2,1	2117	2117	0			1	1		3,5
NB/GNBN	2456	2,1	2697	2697	0		2	20	4		9,3
NB/GNBN	0	1	2303	2303	0			1	1		2
NB/GNBN	111	1	1760	1760	0		2	1	1		4,6
NB/GNBN	366	3	2348	2348	0		2	1	6	1	
NB/GNBN	4	1	1518	1518	0		2	1	3		
NB/GNBN	680	2,2	1783	1783	0		2	1	3		7
NB/GNBN	824	1	2723	2723	0	0	2	1	3		2,5
NB/GNBN	164	1	2533	2533	0		2	1	1		3
NB/GNBN	5	2,1	2615	2615	0		2	1	3		5
NB/GNBN	875	2,2	1830	1830	0		2	1	3		5
NB/GNBN	3	2,1	2055	2696	2	4	-1	1	1		
NB/GNBN	112	1	113	1029	2	2	2	1	4		
NB/GNBN	337	1	2400	2400	0	0	2	1	1		2
NB/GNBN	4110	3	991	991	0		4	1	1		9,9
NB/GNBN	195	1	2531	2531	0			1	4		
NB/GNBN	601	2,2	2399	2399	0		2	1	1		5,9
NB/GNBN	222	2,1	1955	1955	0		2	1	3		6,2
NB/GNBN	383	3	2535	2535	0		4	30	3		
NB/GNBN	276	3	2464	2464	0		2	1	1		7
NB/GNBN	172	-2,2	1728	1728	0			1	5		
NB/GNBN	65	1	2407	2407	0			1	3		1,8
NB/GNBN	0	1	2525	2525	0	0		1	1		3
NB/GNBN	433	3	2112	2112	0		4	50	3		6
NB/GNBN	2	3	1001	1001	0		2	1	1		
NB/GNBN	0	1	468	468	0		2	1	1		
NB/GNBN	7117	3	554	1199	1	4	1,1	1	3		4,8
NB/GNBN	2540	3	2050	2050	0		2	1	4		8
NB/GNBN	5	3	1602	1602	0		2	1	3		7,7
NB/GNBN	1585	3	483	846	1	4	1,1	1	3		7
NB/GNBN	945	3	1839	1839	0		4	30	1		5,3
NB/GNBN	6	2,1	672	672	0			1	1		3,2
NB/GNBN	6	2,1	249	2016	2	4	2	1	3	0	1,7
NB/GNBN	356	2,2	2390	2390	0	0	2	1	1		6,2
NB/GNBN	778	2,1	2044	2044	0		2	1	1	1	4

NB/GNBN	118	2,1	1039	2248	2	4	2	1,5	1		
NB/GNBN	224	1	2743	2743	0		2	1	1		
NB/GNBN	530	1	2315	2315	0		2	1	1		2,5
NB/GNBN	571	3	2420	2420	0		4	50	1		10
NB/GNBN	633	3	111	2433	2	4	2	1	6		7,8
NB/GNBN	186	1	1513	1513	0		2	1	3		3
NB/GNBN	42	2,1	2054	2054	0		2	1	1		6
NB/GNBN	145	3	2277	2277	0	0	2	1	5		
NB/GNBN	318	1	2666	2666	0			1	4		1,5
NB/GNBN	1	2,1	1765	1765	0		2	1	5		3
NB/GNBN	557	3	184	264	1	2	1,1	20	1		8,5
NB/GNBN	54	3	2276	2276	0		2	1	3		6,5
NB/GNBN	3	1	2390	2390	0	0	2	1	1		
NB/GNBN	765	2,2	2306	2306	0	0	2	1	1		4
NB/GNBN	103	1	2324	2324	0		2	1	1		2
NB/GNBN	11	3	2526	2526	0		-1	1	5		
NB/GNBN	758	1	363	2085	2	2	2	1	1		5,7
NB/GNBN	0	1	2387	2387	0		2	1	1		3,5
NB/GNBN	528	1	1907	1907	0		2	1	3		1,8
NB/GNBN	13	3	2186	2186	0		2	1	6	0	2
NB/GNBN	25	1	1680	1680	0		2	1	1		
NB/GNBN	40	1	2310	2310	0	0	2	1	1		5,4
NB/GNBN	245	3	2410	2410	0		2	15	3		6
NB/GNBN	767	3	330	404	1	4		15	3	1	8,5
NB/GNBN	76	3	2085	2085	0		1,1	10	4	1	10
NB/GNBN	561	3	251	251	1	0	4	20	3		10
NB/GNBN	87	2,1	155	2185	2	4	2	1	1		3,8
NB/GNBN	0	2,1	2041	2041	0		2	1	4		1,9
NB/GNBN	198	-1	203	1538	2	2	1,1	1	1		4
NB/GNBN	256	2,1	1016	1016	0		2	1	3	1	
NB/GNBN	482	2,1	2099	2099	0			1	3		0,9
NB/GNBN	1439	1	2368	2368	0		2	1	4		1
NB/GNBN	301	2,1	2150	2150	0		2	1	6		3
NB/GNBN	312	3	2144	2144	0		2	1	3		9,2
NB/GNBN	249	1	2006	2006	0		2	1	1		2,9
NB/GNBN	2	2,2	1656	1656	0		2	1	5		2,5
NB/GNBN	13	1	2464	2464	0			1	1		3,5
NB/GNBN	2041	1	2280	2280	0	0	4	1	1		7,5
NB/GNBN	1017	3	250	364	1	2	1,1	30	3		5
NB/GNBN	262	1	2017	2017	0		1,1	1	1		3,5
NB/GNBN	111	3	168	1792	2	4	2	1	1		4,4
NB/GNBN	134	2,1	2029	2029	0		2	1	3		5,1
NB/GNBN	330	3	2278	2278	0		2	1	4	1	14

NB/GNBN	121	2,1	2143	2143	0		2	1	3		6,7
NB/GNBN	1701	3	2365	2365	0		2	1	1		
NB/GNBN	167	2,2	2295	2295	0	0	2	1	1		5,5
NB/GNBN	0	2,1	107	1453	2	4	2	1	1		3,4
NB/GNBN	29	2,1	1851	1851	0		2	1	1		6
NB/GNBN	315	1	1663	1663	0		2	1	1		5
NB/GNBN	83	-1	15	15	0		2	1	1		3
NB/GNBN	526	3	295	429	1	2	1,1	15	3		11
NB/GNBN	2111	2,1	2253	2253	0		2	1	1		10,6
NB/GNBN	625	3	2163	2163	0		-1	100	1		4
NB/GNBN	59	1	1976	1976	0		2	1	1		3
NB/GNBN	13	2,1	98	1980	2	4	2	1	4		2
NB/GNBN	0	2,1	70	2170	2	4	2	1	1		
NB/GNBN	1810	2,2	2107	2107	0		2	1	4		0,8
NB/GNBN	74	2,1	182	2105	2	4	2	1	4	1	
NB/GNBN	1004	3	866	1540	1	2	1,1	1	1		
NB/GNBN	133	2,1	2223	2223	0		2	1	3		4
NB/GNBN	385	3	1933	1933	0		2	1	4		3
NB/GNBN	890	2,1	1774	1774	0	0	2	1	6	1	6,5
NB/GNBN	217	2,2	1726	1726	0		2	1	4		6
NB/GNBN	103	1	2114	2114	0		2	1	1		2
NB/GNBN	370	2,2	1597	1597	0		2	1	1		4
NB/GNBN	933	1	1834	1834	0		1,1	1	4		1,9
NB/GNBN	1895	3	307	416	1	4	2	1	3		7,1
NB/GNBN	3181	2,1	295	295	1		2	1	4		4,6
NB/GNBN	1408	3	540	1120	1	2	2	1	1		7,1
NB/GNBN	1221	3	1952	1952	0		4	50	3		7,5
NB/GNBN	433	1	2100	2100	0		4	50	1		3,6
NB/GNBN	1683	3	2014	2014	0		2	1	4		8
NB/GNBN	90	1	1483	1483	0	0	2	1	1		2,9
NB/GNBN	17	3	33	1903	2	4	2	1	6		7
NB/GNBN	379	2,1	10	1772	2	4	2	1	3		8,7
NB/GNBN	463	1	2080	2080	0		2	1	3		4,5
NB/GNBN	64	3	1862	1862	0		2	1	6	1	
NB/GNBN	0	2,1	2037	2037	0		2	1	1		
NB/GNBN	85	3	1919	1919	0		2	1	1	1	
NB/GNBN	614	2,2	109	249	1	2	2	15	1		5
NB/GNBN	732	1	1555	1555	0		-1	1	3		5,5
NB/GNBN	276	2,1	2027	2027	0		2	1	3		9
NB/GNBN	20	1	1989	1989	0		2	1	1		2
NB/GNBN	1798	1	1470	1470	0	0	2	1	1		8
NB/GNBN	443	1	1834	1834	0		2	1	4		5,7
NB/GNBN	300	2,1	1775	1775	0		2	1	4	1	4

NB/GNBN	2253	2,1	306	752	1	4	1,1	1	3		8,3
NB/GNBN	267	1	1831	1831	0		1,1	1	1		3
NB/GNBN	0	1	1912	1912	0		2	1	1		4,9
NB/GNBN	412	3	982	2171	2	4	2	1	1		6
NB/GNBN	1157	3	1179	1179	0		2	1	1		
NB/GNBN	596	2,1	1720	1720	0		2	1	1		2,3
NB/GNBN	5210	3	743	987	1	4	2	1	3		
NB/GNBN	217	3	1860	1860	0		2	1	3	1	6,3
NB/GNBN	246	2,2	322	1786	2	2	2	1	1		5,5
NB/GNBN	1424	3	1811	1811	0		2	1	4		7,1
NB/GNBN	14	2,1	1870	1870	0		2	1	5		
NB/GNBN	3739	2,1	1835	1835	0		-1	1	1		7
NB/GNBN	132	3	1780	1780	0		-1	1	3		2,5
NB/GNBN	42	2,1	83	1930	2	4	2	1	1		3,8
NB/GNBN	224	3	1898	1898	0		2	1	3		6
NB/GNBN	3447	1	1306	1306	0	0	-1	1	3		4,9
NB/GNBN	0	2,2	1691	1691	0		2	1	1		3,2
NB/GNBN	2404	1	1394	1394	0			1	4	1	
NB/GNBN	68	2,1	1661	1661	0	0	2	1	1		1,7
NB/GNBN	23	1	1582	1582	0		2	1	5		1,7
NB/GNBN	20	1	1839	1839	0			1	6		2,3
NB/GNBN	227	3	321	1772	2	2	2	1	3		4,3
NB/GNBN	146	1	1788	1788	0	0	2	1	6		5,1
NB/GNBN	1032	1	1819	1819	0		2	1	4		2,3
NB/GNBN	583	2,2	1079	1079	0			1	1		3,7
NB/GNBN	280	3	1662	1662	0		2	1	3	1	
NB/GNBN	791	1	1903	1903	0		2	1	3		4,9
NB/GNBN	1676	2,2	1791	1791	0		2	1	1		3,5
NB/GNBN	547	2,1	767	1110	5	5	-1	1	3		5
NB/GNBN	332	2,1	1629	1629	0		2	1	3		
NB/GNBN	3176	3	1762	1762	0		2	1	3		5,6
NB/GNBN	19	1	1879	1879	0			1	3		2,1
NB/GNBN	75	1	1958	1958	0	0	2	1	1		3
NB/GNBN	5	3	1624	1624	0		-1	1	4		8,8
NB/GNBN	732	2,1	1069	1069	0		2	1	1		
NB/GNBN	27	1	1494	1494	0		2	1	5		
NB/GNBN	127	3	1939	1939	0		2	1	4	1	4
NB/GNBN	29	1	1866	1866	0		2	1	1		
NB/GNBN	569	2,2	1299	1299	0		2	1	6		5,6
NB/GNBN	409	3	450	1570	2	4	2	1	3	1	7,2
NB/GNBN	707	3	1282	1282	0		2	1	1		6
NB/GNBN	198	2,1	1962	1962	0		2	1	3	1	6,6
NB/GNBN	265	2,1	77	1963	2	4	2	1	1		7

NB/GNBN	152	3	75	1847	2	4	2	1	1		5,5
NB/GNBN	337	2,1	1586	1586	0		1,1	1	5		
NB/GNBN	2872	2,1	1192	1192	0		2	1	8		4,4
NB/GNBN	736	1	1220	1220	0		2	1	4		1,6
NB/GNBN	576	2,1	1891	1891	0	0	2	1	5		
NB/GNBN	133	1	1538	1538	0		2	1	1		5
NB/GNBN	2432	1	1681	1681	0		2	1	1		2,5
NB/GNBN	165	1	1086	1086	0		2	1	1		
NB/GNBN	363	3	1910	1910	0		2	1	3		7,8
NB/GNBN	0	1	1717	1717	0		2	1	1		4,2
NB/GNBN	98	1	1637	1637	0		1,1	1	1		1,9
NB/GNBN	259	1	715	1854	2	2	2	1	3		2,7
NB/GNBN	581	2,1	172	1680	2	4	2	1	3		3,5
NB/GNBN	53	1	1565	1565	0		2	1	1		2,8
NB/GNBN	101	1	1397	1397	0		2	1	1		
NB/GNBN	566	1	1442	1442	0		2	1	1		1,8
NB/GNBN	226	1	1658	1658	0	0	2	1	5		4
NB/GNBN	118	1	1546	1546	0		2	1	1		2,5
NB/GNBN	129	2,1	1823	1823	0		2	1	1		2,6
NB/GNBN	51	2,2	1491	1491	0		2	1	5		
NB/GNBN	227	3	112	1540	2	4	2	1,5	3	1	
NB/GNBN	1	3	150	1524	2	4	2	1	4	1	6
NB/GNBN	218	3	1354	1354	0		2	1	1		6,4
NB/GNBN	52	2,1	1756	1756	0		2	1	1		2,9
NB/GNBN	2225	2,2	928	1008	2	2	1,1	1	1		5,5
NB/GNBN	9	3	390	390	0		2	1	4		6,8
NB/GNBN	0	1	959	959	0		2	1	1		1,5
NB/GNBN	1914	3	805	1674	1	4	1,1	1	3		10
NB/GNBN	913	3	275	494	1	4	2	15	1		10,5
NB/GNBN	98	1	776	776	0		2	1	3		2,8
NB/GNBN	8	2,1	1605	1605	0		2	1	5		1,3
NB/GNBN	129	1	1624	1624	0		2	1	4		
NB/GNBN	129	2,2	1306	1306	0		2	1	1		4,5
NB/GNBN	3488	1	1492	1492	0		2	1	1		6,5
NB/GNBN	0	2,2	68	1218	2	4	2	1	1		
NB/GNBN	1099	2,2	1044	1044	0		2	1	3		4
NB/GNBN	84	3	1586	1586	0		-1	1	1		
NB/GNBN	10	3	1419	1419	0		2	1	3	1	3
NB/GNBN	262	1	1461	1461	0		2	1	1		2,2
NB/GNBN	0	2,1	1454	1454	0		2	1	3		6,3
NB/GNBN	120	3	1334	1334	0		2	1	3	1	6,7
NB/GNBN	28	3	188	1330	2	4	2	1	1		4
NB/GNBN	428	3	1463	1463	0		2	1	3		9

NB/GNBN	1742	3	1020	1020	0		4	20	3		13
NB/GNBN	2261	3	497	530	1	4	1,1	20	1		10
NB/GNBN	560	1	1389	1389	0		2	1	3		5,6
NB/GNBN	0	3	186	1323	2	4	2	1	1	1	5
NB/GNBN	84	2,1	1312	1312	0		2	1	4		
NB/GNBN	279	3	738	1144	2	4	2	1	1	1	10,5
NB/GNBN	23	1	1441	1441	0		2	1	1		2,1
NB/GNBN	18	1	1324	1324	0		2	1	3		2
NB/GNBN	47	2,1	1495	1495	0		2	1	1		
NB/GNBN	1067	3	692	1426	2	4	2	1	3		4
NB/GNBN	49	3	59	1333	2	4	2	1	3		4,7
NB/GNBN	514	2,2	141	141	0		2	1	4	1	
NB/GNBN	2	2,1	1518	1518	0		2	1	1		6
NB/GNBN	170	2,1	1261	1261	0		2	1,5	5		1,5
NB/GNBN	1516	1	1350	1350	0		2	1	1		
NB/GNBN	1267	2,1	102	1188	2	4	2	1	4	1	4,6
NB/GNBN	356	1	1211	1211	0		2	1	3		2,8
NB/GNBN	999	3	1009	1009	0		1,1	999	1		6,9
NB/GNBN	271	3	1226	1226	0		2	1,5	4	1	5
NB/GNBN	790	3	1198	1198	0		2	40	3		
NB/GNBN	204	2,2	1207	1207	0		2	1	5		1,4
NB/GNBN	34	1	1136	1136	0		2	1	1		2,5
NB/GNBN	448	3	295	1514	2	4	2	1	4	1	4,5
NB/GNBN	3	2,1	390	1239	2	4	2	1	1		3
NB/GNBN	711	3	196	480	1	4	1,1	1	3		8
NB/GNBN	91	1	1152	1152	0		2	1	1		4
NB/GNBN	1057	3	349	1123	1	2	2	1	1		15
NB/GNBN	636	2,1	1559	1559	0		2	1	4	1	
NB/GNBN	121	3	74	1445	2	4	2	1	6	1	7,5
NB/GNBN	410	3	180	180	0			999	1		7,5
NB/GNBN	363	2,1	1340	1340	0		1,1	1	3	1	
NB/GNBN	263	3	1038	1038	0			-1	6	1	
NB/GNBN	56	1	1134	1134	0		2	1	1		2,9
NB/GNBN	310	3	98	103	1	4	4	50	1		6,5
NB/GNBN	38	3	1281	1281	0		2	1	6	1	4
NB/GNBN	1947	1	763	763	0		2	1	1		11,3
NB/GNBN	423	3	417	526	1	2	4	50	1		13
NB/GNBN	1787	2,2	244	1340	2	2	2	1	1		6,4
NB/GNBN	829	3	1313	1313	0		4	70	1		6
NB/GNBN	460	1	1160	1160	0		2	1	1		1,8
NB/GNBN	11	1	937	937	0		2	1	1		4,4
NB/GNBN	316	-2,1	950	950	0		2	1	3		4
NB/GNBN	306	1	1108	1108	0		2	1	1		4,4

NB/GNBN	2087	3	1236	1236	0		1,1	1	3		5
NB/GNBN	695	3	1168	1168	0		2	1	6	1	6
NB/GNBN	948	3	898	898	0		4	30	1		11
NB/GNBN	14	3	1280	1280	0		2	1	6	1	2
NB/GNBN	622	3	754	754	0		2	1	3		6,9
NB/GNBN	860	3	1059	1059	0		2	1	3		2,7
NB/GNBN	679	1	1181	1181	0		2	1	1		
NB/GNBN	751	1	1037	1037	0		2	1	6		7,8
NB/GNBN	34	1	990	990	0		2	1	1		1,5
NB/GNBN	233	3	860	860	0		2	1	1		4,6
NB/GNBN	558	2,1	863	863	0		2	1	3		3,8
NB/GNBN	257	1	848	848	0		2	1	1		2
NB/GNBN	245	1	850	850	0		2	1	1		4,3
NB/GNBN	128	3	254	995	2	4	2	1	3	1	2,2
NB/GNBN	217	3	1028	1028	0		2	1	3		5,6
NB/GNBN	34	1	1105	1105	0		4	10	1		2,8
NB/GNBN	1549	3	1085	1085	0		2	1	3	1	2,1
NB/GNBN	540	-2,1	143	143	0	0	2	1	6	1	
NB/GNBN	20	2,1	1033	1033	0		2	1	3	1	
NB/GNBN	532	1	996	996	0		2	1	3		
NB/GNBN	1852	1	1209	1209	0		2	1	4		5,5
NB/GNBN	599	3	794	794	0		4	20	1		8,7
NB/GNBN	843	2,1	225	409	1	4	2	1	3	1	4,8
NB/GNBN	209	2,1	1054	1054	0		2	1	5		5,5
NB/GNBN	286	3	825	825	0		2	1	3		
NB/GNBN	46	2,2	1009	1009	0		-1	1	5		3
NB/GNBN	5	2,1	827	827	0		2	1	6		3,1
NB/GNBN	437	1	1121	1121	0		2	1	1		4,3
NB/GNBN	734	1	804	804	0		2	1	1		3,7
NB/GNBN	12	2,1	623	623	0		2	1	3	1	
NB/GNBN	3866	3	915	915	0		2	1	3		13
NB/GNBN	39	1	912	912	0		2	1	5		4,9
NB/GNBN	124	2,1	812	812	0		2	1	3		7,7
NB/GNBN	35	2,1	1023	1023	0		-1	1	1		1,5
NB/GNBN	136	2,1	53	976	2	4	2	1	3		11
NB/GNBN	5578	3	858	858	0		1,1	1	4		
NB/GNBN	101	3	907	907	0		2	1	6		
NB/GNBN	589	1	899	899	0		2	1	1		6
NB/GNBN	327	2,1	395	395	0		2	1	3		8,7
NB/GNBN	43	1	856	856	0	0	2	1	1		3,2
NB/GNBN	126	1	339	339	0		2	1	1		2,6
NB/GNBN	5225	2,2	337	966	2	2	2	1	1		16
NB/GNBN	1590	3	765	765	0		2	20	1		11,5

NB/GNBN	80	2,1	423	423	0		-1	1	6		1,5
NB/GNBN	7484	1	1076	1076	0		2	1	3		6,7
NB/GNBN	63	2,2	395	395	0		2	1	3		3
NB/GNBN	238	3	70	997	2	4	-1	1	1		10,4
NB/GNBN	312	3	678	678	0		2	1	3		6,7
NB/GNBN	336	3	850	850	0		2	1	3		4
NB/GNBN	456	3	839	839	0		2	1	3	1	8,8
NB/GNBN	75	1	785	785	0		2	1	1		3,5
NB/GNBN	2333	3	1074	1074	0		4	20	1		4,7
NB/GNBN	1885	3	441	1020	2	4	2	1,5	1		
NB/GNBN	568	2,2	239	694	2	4	2	1	4	1	
NB/GNBN	118	2,1	1010	1010	0		2	1	1		6,3
NB/GNBN	329	1	7	7	0		1,1	1	1		6
NB/GNBN	1533	1	782	782	0		2	1	3		6,1
NB/GNBN	417	2,1	573	573	0		1,1	1	1		5
NB/GNBN	4102	1	877	877	0		2	1	3		7,5
NB/GNBN	122	1	761	761	0		2	1	1		3,9
NB/GNBN	112	2,1	657	1391	2	4	2	10	1		3
NB/GNBN	534	3	931	931	2	4	2	1	3		9
NB/GNBN	86	2,1	999	999	0		1,1	1	1		
NB/GNBN	20	2,1	282	282	0		2	1	1		3,2
NB/GNBN	38	2,1	879	879	0		2	1	3		5,5
NB/GNBN	667	2,1	479	479	0		2	1	1		2,4
NB/GNBN	68	1	423	423	0		2	1	5		2
NB/GNBN	205	3	607	607	0		2	1	3	1	6,5
NB/GNBN	768	3	755	755	0		4	1,5	1		12
NB/GNBN	1197	1	734	734	0		2	1	3		6,4
NB/GNBN	1300	1	217	217	0		2	1	4		6
NB/GNBN	0	2,1	380	380	0		2	1	1		3,2
NB/GNBN	757	2,1	595	595	0		2	1	3		1,4
NB/GNBN	19	1	629	629	0		2	1	1		4,3
NB/GNBN	73	1	691	691	0		1,1	1	6		4
NB/GNBN	30	1	282	282	0		2	1	1		3,3
NB/GNBN	4	1	820	820	0		2	1	1		3,6
NB/GNBN	71	2,1	794	794	0		2	1	1	1	

TU_BREIT	TU_LANG	VOLUMEN	IBG_PT	HVA_U	HVA_U_F	VMA_U	VMA_U_F	NSE	NSE_WERT	ZUFALL	TUMORSCHW
8,5	11,4	237,4	2	3		9		2	21,1	1	0
3,5	5	35	3	9		9		1	16,4	1	0
5,3	9,1	142,2	9	2	2,31	2	1,43	2	16,2	0	0
7	10	280		2	1,17	9	0,62	3		0	0
13,4	11,3	1022	9	2		2		0		1	0
										0	0
3	4	24	9	2		9		2	17,52	0	0
10	22	1100	3	2		9		2	28,4	1	1
12	16	768	9	2		9		1	16	1	0
4,6	7,6									0	0
3,5	6	36,7	3	9		9		3		1	0
4	5	30	9	9		9		9	18	1	0
6,6	11	280	0	9		9		0		1	0
			9	9		9		2	32,3	1	0
9,5	10,5	419	9	2		9		3		1	0
6	8,5	178,5	2	2		2		2	36,4	0	0
5,4	5	156,6	9	9		9		2	21	1	0
7,8	13,4	541	9	9	0,98	9	0,54	9	8,4	1	0
9,7	15,5	880	9	1		9		1	20,2	1	0
3	3	13,5	9	9		2		2	34,8	0	0
5,6	6,7	118,2	3	3		3		3		1	0
6	8	120	2	2	3,82	2	7,02	2	32,8	1	0
7	7	110	9	2	4,11	2	5,11	9	19,2	1	0
2,3	4,3	18,8	9	9		9		9		0	0
3,6	5,4	65	9	9	0,96	9	0,64	9	13,2	1	0
										0	0
5,5	5,5		3	9		9		2	23	1	0
3,9	5	34,1	3	9		9		3		1	0
2	0,8	1,6	9	2		9		2	24	0	0
11,9	13,7	815,2	2	2	2,65	9	0,71	3		0	0
3	4	27	2	2	2,2	9	0,91	9	12,9	1	0
3,6	4,3	36,4	2	9		9		0		1	0
4	8,5	85	9	9	0,3	9	0,38	9	4,9	0	0
7,5	10,5	244,1	3	2	1,1	9	0,7	9	13,5	0	0
			2	2	2,2	2	2,1	9	19,2	0	0
5,5	5,9	61,66	9	9		9		9	10,9	0	0
2,4	3	7,6		9		9		2	38,6	0	0
2,2	3,5	22,7		9	0,47	9	0,87	9	20	1	0
10	10	700	9	9		9		9		0	0
5	5	75	9	9		9		9	18	0	0
5,5	7			9		9		2	17	0	0
5,8	7,5	69,6	2	2		2		3		0	1

5	5			0		0				1	0
3,9	9,4	84,3	3	9		9		0		1	0
6	8	72	9	9		9		1	16,7	1	0
4,7	4,5	32,8	3	9		9		9		1	0
15	18,5	1100		2		9		2	16,3	1	0
4	7,5	57	9	9	0,87	9	0,88	1	23,9	0	0
4	8	128	9	9		9		9		0	0
10	10	400	9	9	0,22	9	0,17	3		0	1
4	5		3	9		9		9	18,3	0	0
2,8	9,5	65,2	9	9	0,54	9	0,51	9	19,8	1	0
9	10	360	9	9		9		9	11,9	1	0
5	6	60	9	9		9		1		0	0
2,6	4,1	25,1	2	2		9		9	15,5	0	1
		192	9	2		2		9	15	0	0
12,5	10	675	9	9		9		3		1	0
8,5	11,5	464,3	9	9		9		9	11,1	1	0
3,6	3,6	23,3	2	2		2		9		0	1
3	3	6,75	2	9	0,51	9	0,48	0		1	0
8	6	264	9	9		9		9	20,4	0	0
2,2	6,3		9	9	0,36	9	0,29	2	49,7	0	0
6	6		9	9	0,35	9	0,37	9	24	0	0
2,6	3,4	27,8	3	9	0,77	9	0,78	2	25,6	0	0
3,5	4,2	16,1	3	9		9		9	13,9	1	0
12,5	14	831,3	2	2	8,1	2	2,6	9	13,7	1	0
5,3	5,6	75		9		9		9	21,5	0	0
6	7	168						2	37,6	1	0
4,7	9,8	145,1	3	9		9		3		1	0
3,8	6,6	45	9	9		9		2	27,9	1	0
3	2,5	6	9	9		9		9	15,1	0	0
9	7	252	0	2		2		2	20,5	1	0
3	4	15	2	2	1,3	2	1,3	9	15	0	0
4,2	3,2	28		9		9				0	0
5	3,5	54,5	2	9		9		3		0	0
6	10	180	2	9		9		9		1	0
3,5	5	26,25	0	9		9		3		0	0
3,5	5,5	14,5	9	9		9		9		1	0
3,5	4	21	3	3		3		9	15,3	1	0
7,5	9,3	240,6	9	2	1,5	2	1,5	2	29,3	1	0
4,5	5	106,9	9	9		9		2	26	1	0
8	10,1	290,8	3	3		9		2	13,3	0	1
4	7,5	67,5	3	9		9		3		1	0
2,9	4,1	30,3	9	9		9		9	11,7	0	0

9	12	378	0	2	1,41	9	0,87	9	8,9	0	0
4,8	6,5	56,2	9	9		9				1	0
2,5	4,5	12,4	2	2	2	9				0	1
9	14	504	9	2	3,88	9	0,7	3		1	0
5	6	90	9	9		9		9	9,2	0	0
4	4	24	9	9		9				0	1
2,7	3,2	20						2		0	0
4,3	6	61,92	9	9		9		9	16	0	0
4,8	9	86,4	1	0		0		2		0	0
7	8	87		9		9		9		0	0
4,7	5		3	9		9		3		1	0
7	5		9	2		2		3		1	0
6,6	9		9	2	3	2	2	9	19,82	0	0
6,2	7,3	88	9	9		9		9	14,1	0	0
8,5	8,5	155	2	9		9		9	24,2	0	0
5	3	30	9	9		9		9	13,5	0	0
2,6	4,4	13,1	9	0		0		0		0	1
5,1	10,5	80,3	9	9	0,76	9	0,31	2	30,9	0	0
8,5	8,5	170	9	2		9		9		0	0
2,6	3,3	14,5	3	9		9		2	17,6	0	0
7,2	6,3	150	2	3		3		9	10,7	1	0
10	10	450	2	9	0,99	9	0,51	9	7,8	0	0
8	9	288	2	2	4,28	2	2,52	2	16,1	0	0
15	12	900	9	9		9		2	39	0	1
			9	9		9		2	21,6	1	0
2	8	40	9	9		9		9	6,5	1	0
4	4	35	9	9		9		9	6	1	0
4,3	6,6	75	9	2	1,07	2	1,2	9	13	0	0
2,6	4,6	15	3	9		9		2	14,3	0	0
3,4	5,7		9	9	0,81	9	0,45	9	19,7	0	0
7	12	210	9	9	0,45	9	0,38	2	18,9	0	0
										0	0
			2	9		9		9	9,1	0	0
13	11	680	3	9		9		9		0	0
9	13	409	3	9	0,28	9	0,18	9	14,6	0	0
5	5,7	114	9	9		9		2	19	0	0
			3	9		9		2	17	1	0
7,8	7,7	282	9	2		2		2	14,13	1	0
4	4	32	9	2		9		9	8,29	0	0
3,7	6,5	90	2	2		9		9	20,3	0	0
4,5	5	33,8	9	9		9		2	38,4	0	0
3,8	4,2	44,7	9	2		2		3		0	0
8,3	13,1	326	9	2		2		2	35	0	0

2,5	4	9	3	3		3		3		1	0
3	4	19,2	3	3		3		9	16	1	0
			9	9		9		9		1	0
4	5	56	9	9		9		2	24,6	1	0
4	4,5	34,2	1	2		9		1	25,7	1	0
7	10	140	9	9	0,83	9	0,81	2	21,1	1	0
6,5	7,5	105	2	2	3,77	2	16,06	9	13,5	0	0
7,7	9,2		9	9		9		9	11,8	0	0
3,5	4	18,2	3	9	0,5	9	0,64	9	21	1	0
2	5,5	21,7	9	9	0,31	9	0,54	9	16,2	1	0
7	5	157,5	2	9		2		2	23	1	0
8,4	7,5	768,6	3	9		9		3		0	1
2,4	5	30	9	9	0,48	9	0,42	9	19,7	1	0
6	6	72	0	9		9		2	16,7	0	0
3,5	3,5	12,3	3	9	0,46	9	0,17	2	18,2	1	0
4,5	8,5	66,9	9	9		9		2	34,9	0	1
			9	2	1,36	9	0,91	2	23,3	1	0
3,3	5,7	40,9	9	9		9		2	35,5	0	0
3	9	25,7	9	9		9		2	37,2	0	1
11	13,5	742,5	9	2	2,86	2	1,88	1	18,7	0	1
										0	0
5,2	6,7	174,2	2	2		2		2	33	0	0
4	6	96	3					9	11,4	1	0
5,5	6,5	160,8	9	9		9		3		0	0
2,9	5,6	16,2	2	9		9		9	19	0	0
			9	2		2		2	23,7	0	1
6,3	6	160,6	3	2		2		9	8,4	1	0
3,6	2,8	21,2	9	9	0,37	9	0,61	2	28,6	1	0
6	7	147	9	9	0,51	9	0,55	9	12,5	0	0
2,5	3,1	6,6	3	9		9		2	24,8	0	0
6,5	9,6	196	2	2	3,12	2	1,92	3		0	0
2,2	1,6	8,6	9	9	0,49	9	0,9	9	14,9	1	0
3	5	17	9	9		9		2	18	0	0
4,2	5,5	26,6	9	9		9		9	19	0	0
3,3	5,5		2	2	1,09	9	0,73	9	14,5	1	0
3	1	1,5	9	9	0,52	9	0,41	9		0	0
3	5	30	3	9		9		2	22	1	0
7,3	6,3	243	2	9	0,89	9	0,53	2	23,2	1	0
2,2	3,3	9,4	2	9		9		2	21	1	0
5	6	165	9	2		9		9		0	1
4,5	6,6	63,8	9	1		9		9	10	0	0
3,2	3,6	8,2	2							1	0
3,1	1,6	4,6		9		9		9	10,6	1	0

3,5	3,6	20,2	2	2		2		3		0	0
8	11		2	2	1,91	2	1,49	9	9,8	0	0
16	16,5	594	2	2	5,7	2	3,6	2	25,6	1	0
4	5	40	2					2	30,9	1	0
4,5	5,8	49,6	3	2		2		9	17,3	0	0
7,5	7,5	160	9	2		2		2	22	1	0
2	12	72	3	3		9		2	12,6	1	0
4,7	5,5	56,9		9		9		2	41,1	1	0
3,3	3,7		2	1	0,84	2	1,27	1	19	0	0
			3	9	0,18	9	0,21	3		1	0
6,3	6,6	170,5	9	2		2		9	14,2	0	1
3	3,4			9	0,54	9	0,84	3		1	0
3,5	4	17,5	9	2	1,43	9		2	23,9	0	1
								9		1	0
4,2	5	42	2	2		9		9	14	1	0
			2	2	1,6	2	1,25	2	38,8	0	0
3	4,5	13	3	9		9		9	14,3	0	0
6	8,5	102	2	3		3		3		1	0
12,5	9	871,9	9	2		9		0	14,8	1	0
3,6	5,5	43,6	1	2		2		3		0	0
7	8,5	163,6	3	2		9		9	15,2	1	0
			0	9		9		9	9,6	0	0
4	4	48	9	2		2		3		1	0
			9	9		9		9		0	0
										1	0
5	7	52,5	0	0		0		0		0	1
4,4	4,6	38,5	2	2		2		2	9,5	0	0
4	7	28	2	2		2		9	16,7	1	0
2,5	4,5	11,3	3	3		3		3		0	0
3,6	3,8	17,1	2	9		9		2	23,1	0	0
2,4	3,5	8,4	2	9		2	1,3	2	22,1	1	0
6	5,5	132	3	9		9		2	13	1	0
		290	2	2	7,87	2	3,72	2	38,5	1	0
5	11	240	2	2		2		3		1	0
4	9	63	9	2		2		2	37,1	1	0
5	9,1	97,8	2	2		2		2	21,9	0	1
3,9	9,2	64,6	9	9		9		9	12	1	0
			2	2	2,3	2	1,6	2	26,4	0	0
5	9	157		2	4,96	2	6,69	2	30,8	0	1
1,7	2,8	10,9	9	9		9		9	12,9	1	0
4,1	4,5	35	3	9		9		9	12,5	1	0
8	5	140	3	3		3		3		0	0
5,7	9,1	339,8	2	2	3,8	2	3,8	2	49	0	1

7,5	14	315	9	2		2		2	23,4	1	0
			3	2		2		3		0	0
5,7	10,5	149,6	2	2	1,46	2	1,56	2	24	0	0
3,5	4,5	19,7	3	9	0,68	9	0,79	9	18,9	0	0
6	8	96	2	2	2,86	2	3,46	9	61,6	1	0
5	5,5	34,4	2	9		2		2	15,3	1	0
1,5	1,8	1,9	9	9		9		2	29,6	0	0
1,5	1,7	4,6	2	2		2		2	42,4	0	0
2,9	6		2	2	1,38	2	3,27	2	22,2	0	1
7	9	78,8	2	2	4,5	2	5,97	2	125,6	0	0
2,6	3,2	7,5	2	9		9		2	28	0	0
5	5	62,5	2	2		2		2	24,9	0	0
4,5	6	36	3	9		2		9	46,6	1	0
2,6	3,4	21	2	9	0,56	9	0,91	2	39,8	0	1
		12,4	2	2		2		2	33	1	0
3,8	3,2	28	2	2		2		2	57	1	0
1,7	3	8,2	1	9		9		2	34	0	0
5,4	5,7	78	2	2		2		2	176,7	0	1
6	4		2	2		2		2	57,1	1	0
1,8	1,8	3,2	2	9		9		2	39,9	1	0
2,9	3,2	20	2	9		2		2	23	0	0
2,7	3,8	15,9	2	2		2		3		1	0
2,3	3,1	7,8	3	9		9		9	19,5	1	0
			0	2		2		2	68	0	1
2,8	3,6	11	3	2		2		3		0	0
3,5	3	25	1	2		2		2	36,1	1	0
3,2	3,3	25	3	9	0,58	9	0,77	2	21,2	1	0
4,5	5,5	55,6	3	2		2		0		1	0
4,8	6	74,9	2	2		2		2	63,9	1	0
1,4	3,8	4,7	3	3		3		3		0	0
3	3	13						2	23,9	0	1
			9	2		2		2	15	0	0
7,3	9,1	239,1	2	2		2		9	19	0	1
2,4	2,3	7	3	9	0,72	9	0,55	3		0	0
5	6		2	9		9		2	214	0	0
1	4	6	9	9	0,52	9	0,31	1	13,6	0	0
1	1,7	2,8	2	9		9		2	22,8	0	0
		75,3	2	2		2		2	61	0	0
5,3	5	94	2	9	0,96	2	2,04	2	122,3	0	1
9,5	11	564,3	2	2		2		2	54,8	0	0
6	3	54	3	3		3		2	21,4	0	0
1,2	4,6	3,3	2	2		2		2	27,1	0	0
1,6	1,5	1		2		9		2	15,3	0	0

2,7	4,7	15,3	2	2	2	2	3	2	42,2	1	0
3,1	3,2	18,4	3	2		9		2	28	1	0
			2	9	0,33	9	0,31	2	28,9	0	0
11,4	19	747,3	2	2		9		2	2054	0	1
11,6	15	957	2	2	3,36	2	7,17	2	399,2	0	0
6,8	7,4	111	2	2		2		2	77,9	1	0
			3	9	0,59	9	0,72	9	17,1	1	0
2,4	2,7	5,8	2	9		9		2	28,5	1	0
4	4,8			9	0,53	9	0,24			0	0
2,1	3,5	4,4		9	0,4	9	0,48	9	13	0	0
										1	0
7,1	9	180	2	5	0,99	2	3,08			0	1
4,6	4,2	40	2	9	0,93	2	1,55	9	10,8	1	0
2,5	3,5	10,9	2	2		2		2	22	1	0
		46	2	2		2		1	15	1	0
5	8	100	2	2		2		2	40,6	1	0
2	2	4	2	9		9		2	28,4	1	0
6	6	72	2	2	1,7	2	2,73			1	0
8,5	8	238	2	2	2,64	2	4,44	0		1	0
5,2	6,3	90	2	2		2		3		1	0
5,5	6,5	70	0	9		9		2	34,5	1	0
3,2	2,8	15,7	2	9	0,73	2	1	1	20	1	0
7,5	9	170	9	9		9		2	53,9	0	0
2,1	2,7		2	2		2		9	21,4	1	0
3	3	6,75	2	2		2		2	52,8	1	0
4,4	5,7	62,7	2	2	2,8	2	2,1	2	30,4	1	0
4	4,4	35,2	9	9		9		9	12,35	1	0
5	6	75	2	2		9		2	23,7	1	0
4	5	20	2	2	3,68	2	25,36	3		1	0
3,8	6	46,7		2		2		2	29,2	0	0
3,5	6	47,3	2	2	1,56	2	1,91	2	81,2	0	0
4,8	6,2	81,8	2					2	27,7	1	0
8	6	144	2	2	4,9	2	6,8	2	62	1	0
5	5,5	57,8	2	2	3,78	2	8,03	2	31,3	1	0
3,4	4,6	32,8	2	2	1,56	2	1,19			1	0
5	5	62,5		2		2				1	0
			3					9		0	0
3,5	5	17,5		2		9		9	13,9	1	0
7	6	130	2	2		2		9	13,9	0	0
5	6,5	100	2	9	0,83	2	1,22	3		0	0
1,5	3,6	10		2	1,62	2	1,94	9	15,4	1	0
1,8	3,6	4,9		9	0,92	2	1,29			0	0
1,8	2	2,7	3	9		9		2	44,2	1	0

3,9	5	35,1		2		2		2	36,65	0	0
4	4	33,6	2	2		2		9	21,3	0	0
5,5	5	59,1	3	2		2		2	60,3	0	1
4,3	3,1	25,33	2	9	0,54	9	0,49	2	130	1	0
				2	4,93	2	5,71	2	52,3	0	0
4,1	5,2	40,5	9	9		9		9	9,3	1	0
2,5	3,2	18	3	9		9		2	35	0	0
5	5	46,2	2	9		2		2	21,3	1	0
3,5	2,8	19	2	9		2		1	20	1	0
4,3	5,3	20,6	2	9	0,9	9	0,79			1	0
6	6	153	2	2	5,27	2	3,83	2	35	1	0
4,4	3,6	23	2	1	0,48	1	1,44	9	13,2	1	0
4	5	45	2	2	3,8	2	1,5	0		1	0
5	5	87,5	9	2		2		9	19	1	0
2	2,2	4,8	2	9		9		9	8,9	1	0
6	7	126	2	2		2		3		0	0
										1	0
	3,5	24								1	0
4,3	4,7	49,5	2	2	1,78	2	3,96	9	15,2	1	0
3	3,3	14	2	9		9		9	22,2	1	0
2,2	4	7,5	2	9	0,69	9	0,88	9	19,7		
6	6	54	2	2	2,2	2	2	9	16,7	0	0
3,5	5,5		2	2				2	92	1	0
3,2	3,3	15,3	2	2		0		2		0	0
6,5	11,5	242	2	2	2,86	2	5,02	2	75,1	0	1
2,3	4,2	16,4	2	2	1,01	9	0,79	9	17,4	1	0
			2	2		2				0	1
			3	9	0,96	2	1,12	2	68	0	1
				2		2		9		1	0
			2	2		2		2	370	0	1
2,2	3,5	8,5	2	2		2		9	11,2	1	0
9,5	12,5	385	2	2	25	2	25	2	24,2	0	1
2,9	4,5	17,6	2	9		2		2	31	1	0
1,3	3,5	7,5	2	9	0,51	9	0,43	2	99	0	0
8	12	336	2	2	2,06	2	4,21	2	186	1	0
4,5	5,5	43,3	2	3		3		2	36,4	0	1
2,7	3,5	9,45	2	2	1,8	2	4	2	17,3	1	0
11	11	726	2	1	0,99	9	0,62	2	864	0	0
			2	2		2		2	157	0	1
7,9	6	130,4	2	2		2		2	114	1	0
3,3	3,1	20,5	2	2	1,08	2	1,79	2	33,1	1	0
3,4	8,5	46,2	2			2		2	70,7	0	1
2,6	3,9	20,3	2	9		9		9	21,3	1	0

12	24	1500	2	2	2,35	9	0,42	3		0	0
3,5	4,5	23,6	0					9	14,6	1	0
2,5	4,2	15,8	9	2	2,76	2	5,03	3		0	0
9,1	7,2	147,4	2	2		0	0	2	44,6	1	0
2	6	15	9	2	1,14	2	1,56	9	15,1	1	0
5,8	6,2	111,5	2	2	2,31	9	0,42	3		1	0
6	2,5	52,5		2	1,8	2	1,53	2	34,9	1	0
5,5	13	257,4	2	2	9,31	2	40,27	2	86,8	0	0
2,5	2,5		2	9	0,5	9	0,85	9	9	1	0
			2	2		2		2	56,1	0	1
		7,5	2	9		2		9	9,9	1	0
4,2	6	63	2	2		2		2	54,7	0	1
3	4,7	42,3	2	2	1,86	2	2,52	2	33,8	1	0
11,5	13	710,1	2	2	5,9	2	5,4	2	1047	0	0
8	7	280	2	2		2		9	15,5	0	1
										1	0
3,5	1,1	7,3	2	9		2		2	32,5	0	0
4,9	5	55,1	2	2		2				1	0
4	5,7	45,6		9	0,79	9	0,45	2	30,7	0	1
4	4,1	35	0	2	1,5	2	2,89	2	48,9	1	0
5,5	5,1	74	3	9		9		2	70,2	1	0
2,2	4	30,8	2	2		2				1	0
5	6	45	2	2	1,14	2	1,74	2	42,1	1	0
1,7	4	3,5	2	9	0,36	9	0,69	2	20,3	0	0
			9	2	1,14	2	1,16	2	28,8	1	0
4	6	30	2	2	1,48	2	1,46	3		1	
5,5	4,5	86,6	3	2	1,46	2	1,02	9	14,7	0	1
4	4	24	3	9		1		2	61,7	1	0
5	4		2	2	1,49	2	1,74	2		1	0
3	6	45	2	2	1,21	9	0,95	2	70,2	0	1
3	2,5	15	2	2		2		9	20,6	0	1
7	8	112	2	2	2	2	3,6	2	34,7	0	0
1,5	3	5,62	2	9	0,67	9	0,74	2	66	1	0
			2	9		9				1	0
2,1	2,1	6,6	2	9				9	15	0	0
4,9	6,3	75,6	3	9		9		2	69,1	0	0
2,3	2,7	8,7	2	9		1		2	16,9	1	0
4,6	5,6	74,7	2	2	2,5	2	2,5	2		1	0
10	12	600	2	2	2	2	4	9	20,9	0	1
5,5	9	99	2					2	67,3	1	0
3,2	3,2	15,36	9	9	0,31	9	0,53	3		1	0
10	5	125	2	2		2		2	160	0	1
2	5,5	20		9		2		2	61,2	0	0

6,2	7,3	92,8	2	2	1,48	2	2,65	2	63	0	0
5,2	5,5	82,9	2	2	1,72	2	2,32	2	32,6	1	0
8,5	8,3	246,9	2	2		2		2		1	0
6	8,4	226,8	2	3		3		2	38,9	0	1
7,5	9	219	2	2		2		2	152,5	1	0
13	7		2	2		9		2	617	0	1
6	7	210	2	9		9		9		0	0
4	4	44	2	2		2		2	30	0	0
10	13,5	573,75	3	2		2		2	34	0	0
4	3,7	29,6	2	2		2		2	29	1	0
				0		0				1	0
4	5,5	55		9	0,25	9	0,25	2	28	0	0
2	4			9	0,5	9	0,73	2	51,1	0	0
4,5	5,5	49,5	2	2	1,77	2	3,4	2	97	0	1
3,1	4,4	32	3	9	0,71	9	0,85	9	24	1	0
7,2	8,3	269	3	9		9		9		0	1
6,3	4,6	66,7	2	2		2		2	28,3	0	0
4	3									0	0
6	8	144	2	2		2		2	107	0	0
4,5	7	55	2	9		9		9	8	1	0
4	6	60	2	2	2,38	2	3,17	3		1	0
3	3,5	13,1		9		9		2	26,6	1	0
4	5	50	2	2	2,13	2	1,53	3		1	0
7	6,5	159,25	2	2		2		2	54,1	1	0
1,9	3	7,1	9	9		9		2	50	1	0
4,2	2,8	20,6	2	9	0,95	2	2,49	2	70,2	1	0
4,8	6,6	71,28	2	2		2		2	56	1	0
		800	2	2		2		3		0	1
3,8	4,5	51,3	2	2		2		2	36,7	0	1
4,4	5,6	44,35	2	2		9		2	24,1	1	0
7	9,6	154,6	2	2	13,84	2	5,42	2	63,4	0	0
3,5	4,5	19,7	2	9		9		2	22	0	0
3,8	4	36,5	2	9		9		2	41	1	0
4,7	5,9	74,87	3	3		3		1	26,1	1	0
2,8	3,2	15,2	2	9		2		2	47,6	1	0
				3		3				0	1
5	3	22,5		9		9		3		0	0
9	13	526,5	3	2		9		2	512	0	1
10	10	400	9	9		9		9		0	0
6,1	7,3	129,1	3					2	35,5	1	0
5	5	43,75	2	2	4,75	2	2,99	9	16,3	0	0
6	5	75	3	9	0,51	9	0,69	9	11,4	1	0
1,46	2,8	4,95		9		9		9	11,4	0	0

2,2	2	5,5	2	2		2		2	107,6	0	1
2,1	2,5	3,15	9	9		9		2	30,9	1	0
			3	9	0,41	9	0,82			0	0
5	7	87,5	2	2		2		3		1	0
11	13	780	3	9	0,4	9	0,48	2	143	0	1
3,31	3,64	17,1		9		9		9	16,9	1	0
13	16	364	2	2		2		3		0	0
6,3	6,4	121	2	9		9		2	138,1	0	0
4	6	36		9		9		9	13,8	0	1
3,9	6	70,2	2	2		2		2	125	1	0
6	7	126	2	2	2,4	9	0,78	2	182,1	0	1
7,1	7,6	134,9	2	0		0		2	33,6	0	1
6	6	90	2	2		2		2	54,8	0	1
		600	2	9		9				0	1
2	2,5		3	9		9		9	7,3	1	0
			2	9		9		1	20,2	0	0
2,5	1,8	5,4	2	1	0,96	2	1,16	2	41,3	0	1
1,2	3	1,8		9	0,37	9	0,79	2	68	0	0
4	4	24		3		3		3		1	0
			9	9		9		2	37,66	0	0
4,5	3,6	41,31	2	2	2,73	2	2,23	3		0	0
3,5	3			2		2				0	0
5	6	50	2	9	0,69	9	0,47	1	14,5	1	0
5,6	8,7	131,5	2	2	3,25	2	4,63	2	106,5	1	0
13,5	12	1263	2	2		9		2	183	0	1
8	13	416	2	9	0,26	9	0,37	2	273	0	1
			3	2		2		2	246,7	0	0
3,3	2,4		2	0		0		0	26,6	1	0
2	2	5	2	9	0,3	9	0,29	9	12,3	1	0
7	7	122,5	2	2	2,08	2	5,85	2	47,5	0	1
			2	2		2		2	24,21	0	0
6,7	6,8	164	2	9	0,64	9	0,54	2	41,7	0	0
3,5	2,5	12	3	9		9		3		0	0
3,8	3,5	15,3	2	9		2		2	27,6	1	0
1,8	2,2	5,35		9		9				1	0
5,8	6,1	76	9	9		9				0	0
6,6	10	260,7	2	2		2		2	86,57	1	0
5,3	3,8	38	2	2	1,25	2	1,98	9	18,4	1	0
4	64,8	42	2	2	3,86	2	1,65	2	24,1	1	0
6	5	105	0	9		9		2	35,6	0	1
3,7	4,4	22	2	2	2,4	2	7	3		1	0
2,2	2,8	4,93	3	9		9		3		1	0
			2	2	1,0[illegible]	9	0,42	2	109	0	0

2,4	2,6	5,62	2	9		9		2	19,7	1	0
4,9	4,2	60,7	0	9		2	1,09	2	24,1	1	0
5,6	6	75,6	2	2		9		2	83	1	0
3,5	5	52,5	9	9		9		2	115	0	0
			2	9		9				1	0
			2	9		9		0		0	1
5	6	75	2	2		2		2	41,2	1	0
3	3	7	2	9	0,78	2	1,04	2	28	1	0
4	5	35	2	2		2		9	16	1	0
7,7	10,2	365,2	2	2	1,8	2	1,98	2	11,7	1	0
2	2	4	2	9		9		9	16	1	0
3,6	3,7	30,6	2	2		2		0		1	0
			2	1	0,98	2	1,29	1		0	0
				2	2,5	2	3	2	72,6	1	0
9	7	220,5	2	2	4,52	2	5,25	3		0	1
3,5	4	17,5	2	0		0		2	34,5	0	0
5,4	5,5	44,6	9	9		2		3		0	0
8	7	140	3	9		2		2	70	1	0
5	6	75	2	2		2		9	18,1	0	0
1,4	3,1	4,5	2	9		9		2	36	1	0
			2	2		2		9		0	0
2,8	3	16,8	2	9	0,71	9	0,64	3		1	0
10,5	10,2	530	0	2	3,07	2	3,6	2	38	0	1
			9	9		9		9	8,3	1	0
3,6	3,8	40,3	2	2		2		2	22,6	1	0
3,3	4,3	44	2	2	4,2	2	1,67	2	55	1	0
			2	9		9		2	319,5	0	1
8	13	364	2	2	12	2	28	3		0	1
			3	9	0,82	9	0,81	2	33,4	0	0
1,5	1,6	2,2		9		9		2	21	1	0
5	4	30	3	9		9		2	18,8	1	0
5,3	8,2	130,4	9	9	0,96	9	0,28	2	657	0	1
5,6	5,6		2			2				1	0
				9		9		2		1	0
6,5	7	109,2	2	2		9		9	12,44	0	1
9,5	10,5	399	2	2	1,63	2	2,73	3		0	0
4,9	7,3	145	3	2	6,31	2	17,53	2	277	1	0
8	9	252	2	2		2		2	121,7	0	0
6,1	3,4	55	2	2		2				0	0
1,5	2,4	6	3					2	37	1	0
1,8	2,5	3,8		2		9		2	83	1	0
6,8	6,5	137	2	2		2		9	18	1	0
6,6	10	132	2	2	[illegible]	2	5	2	34,3	1	0

			2	9	0,65	9	0,9	2	16,2	1	0
4	4	28	2	2	2,16	2	1,85	2	45,8	1	0
3	4,5	16,9	2	9	0,69	9	0,51	9	12	0	0
7,5	9	337,5	2	2	1,65	9		2	36,1	1	0
5,2	5,7	149	2	2		2		2	96	0	0
5,5	6,5	62	2	2	2	2	4	2	86,7	1	0
5,8	5,7	99	2	2		2		3		1	0
		7,8	2	2		2		3		0	1
1,5	1,5	1,7	9	9		9		2	37,9	0	0
4	4,5	27		9		9		9	11	0	1
10,5	10	446,2	2	9		9		2	370	0	0
6,5	8	169	2	2	1,85	2	4,4	2	112	1	0
3,7	4		0	2	1,43	2	3,37	2	22,5	0	1
5	7	70	2	3		2		2	27	0	0
2,3	2,1	4,8	9	9	0,58	9	0,72	9	12,3	1	0
4	3		2	2	1,5	2	2	2	112,4	0	1
4	3,7	42,2	2	2		2		2	43,2	1	0
4	4,5	31,5	2	9		9		2	26,4	1	0
2,5	3,5	7,8	2	9		9		2	45,3	0	0
5	5	50	2	2	3,27	2	3,22	3		0	0
		25	0	9		9		3		1	0
4,1	6,7	75	3	0		0		2	60,6	0	0
4	9,4	112,8	2	2		2		2	46,5	0	0
5,5	6,5	151,9	2	2		2		2	539,1	0	1
7	6	210	2	2	1,25	2	3,7	2	61,8	0	0
7	8	280	2	9		9		2	421,5	0	1
4	4	30,4	2	2		2		2	14,7	1	0
1,8	4,5	8,1	2	2		2		0	15	0	1
3,4	5,5	37,4	3	-1		0		3		1	0
	5		3	9	0,25	9	0,54	9	11,5	1	0
1,2	2,3	1,24	9	9		9		2	29,1	0	0
1	5		2	9	0,43	9	0,33	9	14,8	0	0
3,5	3,4	18,3	3	0		0		2	16,4	0	0
7,2	6,1	202	2	2		2		2	370	0	0
3,4	3,7	18	2	9		9		2	22,5	1	0
4	3	15	3	9	0,55	9	0,5	9	17,4	1	0
5	4	35	2	9		9		2	59,2	1	0
8	10	300	3	2		9		2	22,9	0	1
8,9	6,2	137,9	2	9		9		2	66,2	0	0
3,5	3,5	21	2	9		9		9	18,9	1	0
5,2	6,4	73,2	2	2	15	2	20	2	66	1	0
5,5	6,2	86,9	2	3		2		2	63,9	1	0
3	6	126	2	2	6,7	2	8,07	2	148	0	0

5	6,6	112	2	2		2		2	37	1	0
		400	2	2	2,49	2	2,38	3		0	0
5,5	5	75,6	2	2		2		2	41,7	1	0
3,4	5,3	30,6	2	2	3,5	2	3,4	2	83	1	0
5	4,6	69	0	2		2		2	38,5	1	0
5	5,6	70	2	2	2,6	2	7	2	25,1	0	0
4	4	12								0	0
7,6	9,4	500	2	2		2		2	859,8	0	1
4,6	6,5	158,5	2	2	3	2	4,8	2	36,5	1	0
5	6,5	65	3	9		9		9	12,9	1	0
4,3	4,5	29	2	2		2		9	24,1	1	0
3	4	12	0	2		2		2	45,5	0	0
2,2	2,73			9		9		2	30,2	1	0
1,6	3,2	2	2	9		9		2	39,8	1	0
			2	2	1,1	2	3	9		1	0
11	8,5		2	2		2		2	97,33	0	1
4,5	5	45	3	2		2		3		1	0
4	6	36	2	2	1,38	2	4,56	2	26	0	1
6	7	136,5	3	2		9		2	45	1	0
4,5	4	54	2	9		2		2	32,3	1	0
1,8	2	3,6	2	9	0,63	9	0,64	2	47	1	0
5	6	60	2	2	3,4	2	5,5	2	38,6	1	0
2	3	6	2	9	0,82	9	0,97	2	31,2	1	0
3,9	7,4	102	2	2							
3,4	7,2	56,3	9	9		9		9	13,9	0	0
14,2	12	631	9	2	10,77	2	20,74	2	200	0	1
8	9	270	9	9		9		2	50	0	0
3,7	4,9	32,6	9	9		9		2	140	1	0
8	9	288	2	2		1		2	49,3	0	0
3	3,2	14	2	9		2		9	7	1	0
7	5	122,5	3	2	3	2	8	2	34,8	1	0
5,3	4,5	103,7	2	2	1,04	2	3,2	2	67,1	0	0
1,8	2,1	8,5	3	2		2		0		0	0
		8,2	3	2		2		2	67,4	0	0
4	4		3	2		2		2	107,8	1	0
			2	2	4	2	5	2	37,1	0	0
9	9	202,5	3	2		2		2	48,7	0	0
3,2	4,3	37,8	3	0		0		0		1	0
8	9	324	2	2		2		2	77	1	0
2	2	4	2	9		2		3		1	0
7	7	196	2	9		9		2	26,4	1	0
3,2	5,1	46,5	2	9		9		2	35,6	1	0
4,9	4,1	40,2	2	2	1,2	2	1,96	2	46,2	0	0

4,7	6	117	2	9		9		3		0	1
3,4	3,7	18,9	2	2		2		9	19,2	1	0
4,2	4,5	46,3	3	9		9				1	0
6	8	100	3	2	10	2	8	3		0	0
			2	9		2		2	53,2	0	0
1,3	1,5	2,2	2	3		9		9	19,1	1	0
		2300	2	3		3		3		0	0
6,5	7,6	155,6	2	2		2		9	22	1	0
4,5	4,2	51,9	2	2		2		2	51,9	1	0
5,8	7,6	176	2	2		2		2	68,3	0	0
			3	9		2		2	45,2	1	0
8,5	9	267,8	9	9		9		2	17,1	1	0
2,5	4	12,5	9	9	0,58	9	0,56	9	13,1	1	0
4	3,7	28,1	2	2	1,6	2	1,6	2	38,5	1	0
7	5	105	2	2	4,7	2	23,6	2	65,6	1	0
3,3	7,7	60,4	9	9		9		2	22	1	0
3,3	3,2	16,9	2	9		9		9	20	1	0
	10		9	9		9		9		0	0
2,1	2,2	3,9	2					9	16,7	1	0
2,5	2,5	10,6	3	9		9		2	30	0	1
2,9	3,4	11	3	3		3		3		1	0
7,5	5,8	93,5	3	2		2		2	47,3	0	1
4,5	7,3	83	2	2	4,93	2	3,46	2	55,1	0	0
2,7	4,2	13	2	9		9		9	16,8	1	0
5,3	5,4	50	2	0		0		1	20,7	1	0
			0	2		2		2	45,3	0	0
6,6	6,6	106,7	2	2	1,29	2	3,34	2	36,2	1	0
6	6,5	76	2	9	0,47	9	0,7	9	7,9	0	0
5,6	7,3	102,2	2	2		2		2	53,5	1	0
4,5	7		3	2		2		2	175,2	0	1
2,2	5	47	2	9		9		9	12,9	0	0
2,1	2,7	5,9	3	3		9		9	15	1	0
3	3	13,5	2	9		9		2	38,9	1	0
6	6	158,4	2	2		2		2	123,9	0	0
			2	2	1,31	2	1,65	2	55	1	0
										1	0
3,7	4,8	35,5	2	2		2		2	11,6	0	0
			2	9	0,74	2	1,88	2	64,6	1	0
6,5	6,2	112,8	3	2		9		2	41,8	0	0
5,7	5,1	104,6	2	2	2,56	2	3,88	2	31,4	0	1
4,5	3,5	47,2	2	2		2		2	22,1	1	0
6,3	8,7	180,9	2	2	7,72	2	13,49	2	65,4	1	0
6	6,5	136,5	2	2		2		2	37,3	1	0

4,2	5,6	64,7	2	2		2		2	109	0	0
		30	2	2	1,15	2	2,6	3		0	1
4,8	4,5	47,5	2	9		9		9	11	0	1
3,5	4,6	12,9	2	9		3		2	32,4	1	0
			2	2		2		2	21,8	0	1
5,1	4,6	58,7	2	2	3,01	2	4,22	9	23,6	1	0
1,5	2	3,75	2	9		9		3		1	0
		4,9	2	9	0,79	2	1,46	9	16,2	1	0
5	7	136,5	2	3		2		2	35	1	0
3,5	2,6	19	2	2	1,85	2	2,93	2	39,4	1	0
3,1	3,3	9,7	2	9		9		9	11	1	0
3,1	5	21	9	9		9		2	50,6	1	0
4	6,6	46,2	9	2	2,87	2	2,68	2	50	1	0
2,4	3,6	12	2	2		2		2	72,3	1	0
			2	2	1,62	2	2,42	2	27,61	1	0
0,6	1,2	0,6	2	9		9		2	42	0	0
3,8	2,7	20,5	2	9	0,67	2	1,19	9	9,3	0	1
2,4	3,4	10,2	2	9	0,67	9	0,6	9	14,8	1	0
2,7	3,5	12,3	2	2		2		3		1	0
		3,5	3	3		3		3		0	0
			2	9	0,92	2	1,37	2	54,3	0	0
6	6	108		2		2		2	81	0	0
6	6,6	127	2	2	2,31	2	5,52	2	71,2	1	0
1,9	2,8	7,7	2	2		2		9	12,38	0	0
6	7,3	115,5	2	2	4	2	3,15	2	32	0	0
3,4	5	57,8	2	2		2		2	27,6	0	1
3	3	6,8	3	3		3		3		1	0
6,5	10	325	2	2	3,26	2	10,4	2	458,5	0	1
7,8	9,5	389	2	2		2		2	377,5	0	1
3,3	3,4	15,7	2	9	0,67	9	0,97	1	20,3	1	0
2,3	4	6	3	2		2		2	87,4	0	1
2	2,5		2	9		9		9	11,9	0	0
6,6	5,6	83	2	2		2		2	62,7	1	0
5,5	7,2	128,7	2	2		2		0		0	0
			3	9	0,38	9	0,83	2	47,1	1	0
4	8	64	2	2		2		2	43,1	1	0
		40	2	2	1,28	2	3,2	2	66,4	1	0
2,2	2,2	7,2	2	2	1,07	2	2,34	2	24,9	0	1
2,8	3	9,2	3	3		9		9	27,9	1	0
3	4,8	45	0	2		2		2	26,3	1	0
4,3	6,3	90,7	2	2		2		2	112,3	0	1
3,4	4,3	29,2	2	9	0,75	2	1,35	9	20,7	1	0
10	11	500	2	2		2		2	318	0	1

10,5	8,8	600	2	2	1,08	9	0,43	0		0	1
6	12	360	2	9		9		2	407	0	0
8,3	8,5	197,5	3	2		2		9	13	1	0
6	7	105	2	2	3,88	2	4,88	2	82	1	0
		81,5	3	2	1,32	2	4,57	3		0	1
10,3	11,2	605,6	2	2	6,29	2	5,7	3		0	0
2,5	2,7	14,1	3	9		2		9		1	0
2	2,5	5	3	9		9		2	24,3	1	0
			2	2	1,35	2	1,89	2	22,7	1	0
2,7	4,7	25,4	2	9		2		3		1	0
5	6,4	76	2	9		9		9	18	1	0
										1	0
4	5	60	0	9		3		2	73,5	1	0
3	6,4	14,4	3	9		9		2	30,3	1	0
		62	2	2	1,8	2	3,64	2	26,6	1	0
7,2	9	149	2	2	6,2	2	4,9	2	30,2	1	0
2,8	3	10,4	2	9	0,47	9	0,54	2	35,3	1	0
9,8	13,8	466,8	2	2	1,25	2	1,02	2	332,4	0	0
4	5,6	56	2	2	1,75	2	2,94	2	46,8	0	1
4	8		2	1		2		2	182,3	0	0
1,6	4	4,5	9	9		9		2	23,5	0	0
1,7	2,6	5,5	3	9		9		9		1	0
5,1	8	91,8	2	2		2	1,48	2	72,5	0	0
3,5	4	21	2	2		2		2	119	1	0
11	12,2	536	2	2	1,99	2	3,32	2	408,5	0	1
4	4	32	2	2		2		9	19,1	1	0
10	9	675	2	2		2		2	761	0	0
			2	2	1,09	2	3,1	0		1	0
4	6	90	3			2				0	1
8	12	360	9	9		9				0	1
3,2	4,4		2	2	1,93	2	1,32	9	20,9	0	0
6	5		2	2		2		2	105	0	0
1,7	2,3	5,6	2	2		2		2	23,4	1	0
11	13	500	2	2		9		2	408	0	1
4,5	8,5	76,5	3	2		2		3		0	1
13	14,7	1080	3	2		2		2	129,5	0	0
9	9	526,5	2	2		9		2	340,1	0	1
7	8	179,2	2	9		9		2	140,3	0	0
9	12	324	2	2	1,27	9	0,58	2	456,5	0	0
3,1	4,4	15		9		9		2	24,7	0	0
4,2	3,4		3	2		2		2	26,5	1	0
3	5	30	2	3		3		9	21,4	1	0
4,8	4,5	47,5	2	2	1,0	[illegible]	3,15	2	84,5	1	0

7	8	140	9	9	0,57	9	0,51	2		1	0
6	7	126	2	2	5,6	2	8,9	3		0	0
10	10	550	2	2	2,99	2	1,97	2	687	0	0
3	6,5	20	2	2		2		2	88,7	1	0
2,7	4,3	40	2	2		2		2	32,95	0	0
3,8	5,4	27,7	2	2	2,32	2	8,1	2	48,4	1	0
1,7	1,8		1	9	0,42	9	0,47	2	22,2	1	0
5,8	5,1	115,4	9	2		2		2	133,7	1	0
2,4	2,3	4,1	2	9		9		9	8,1	1	0
6,9	6,7	106,3	2	2	6,48	2	6,47	2	63,2	1	0
4,1	5,4	42	2	2	1,4	2	3,8	2	66,6	1	0
3,8	3,3	12,5	2	0	0,9	0	1,93	9	18,7	1	0
4,5	5	48,3	2	2		9		2	45	1	0
4,1	6,2	30	2	2	2,7	2	4,7	2	80,9	1	0
10,4	11	320	2	2	29	2	23	2	129,8	1	0
2,7	3,1	11,7	2	9		9		2	87,5	1	0
2,3	6	14,5	9	-1		-1		9	21,4	0	0
										0	0
			2	9	0,76	2	2,1	0		0	1
1,1	1,8		2	1		9		2	34,4	0	0
2,6	4	30	2	2	2,1	2	1,1	2	19,4	0	1
8,2	7,2	256,8	9	9		2		2	195	0	0
4	5,8	55,7	3	3		3		2	94,99	0	0
5	6,5	89,4	2	9		9		2	95,7	0	1
		460	2	2		2		2	150,8	1	0
2,5	3	11,25	2	2	1,38	2	1,48	0		1	0
3,8	3,6	21,2	2	2		2		2	22,7	1	0
4,8	5,3	54,7	2	2		2		3		0	0
4,7	7,3	63,5	9	2		2		2	59,7	0	0
			3	9		2		2	87	0	0
14	10	910	9	2	2,72	2	3,8	2	329,7	0	0
3,6	5,3	50	2	2	23	2	12	2	62,3	0	1
7	9	242,5	3	2		2		2	351	0	1
2,1	2,7	4,2	2	9		9		9	17	1	0
7,3	5,5	220	2	2		2		2	68,9	1	0
		1250	2	2	6,6	2	6,47	2	60	0	1
			2	2	1,5	2	4,7	2	22,1	0	1
6,3	5,2	98,3	9	2		2		2		0	0
6	7,2	187,9	2	1		2		2	54,8	0	0
3,6	3,9	22,4	2	9	0,66	2	1,14	2	45,46	1	0
3,1	3,8	16	2	9		9		2	23,4	1	0
15	17	2040	2	9		9		2	43	0	1
13,3	14	1070	2	2		9		2	510,5	0	1

1,5	2	2,25	3	9	0,52	9	0,53	9		0	0
5,8	7,7	149,6	2	9		2		9	14,6	0	0
2,8	4,1	17,22	2	2		2		9	17,3	1	0
6,3	10,5	343,9	2	2	2,29	2	15,42	2	116,5	0	1
4,4	4,4	64	2	2	1,28	2	2,57	2	88,2	1	0
8	10	150	2	2	4,74	2	6,49	2	93,3	0	1
7	9,2	283	2	2		2		0		1	0
3,5	3,1	19	3	2	1,04	2	2,23	1	21,5	0	0
6,8	6,5	44	2	2	1,76	2	1,1	2	333,1	0	0
			2	2	6,8	2	12,32	2	111,6	1	0
			2	2		2		2	57,1	1	0
5,3	4,8	80	2	2	2,2	2	2,56	2	46	1	0
5	5	75	2	2		2		2	76	1	0
4,9	6	89,7	3	3		2		2	31,1	1	0
4	6	60	2	2	2,3	2	3,1	2	13,7	0	0
8,2	9,7	298	3	3		3		2	20,1	0	0
2,9	4,6	26	2	2	1,33	2	2,36	2	35,5	1	0
3,2	3,8	18	3	2	1,4	2	1,2	3		1	0
6,6	11,2	355	2	2	6,05	2	14,48	2	60,7	0	1
	2,7	10,5	2	9	0,63	2	1,35	9	16,2	1	0
2,7	2,8	12,1	2	2	2,5	2	3,4	2	75	1	0
6,6	6,2	115	2	2	6,34	2	9,49	2	83	1	0
3,9	4	18,7	2	2		2		2	39,4	1	0
1	1,2	1,2	9	9	0,89	9	0,47	2	48	0	0
9	12	351	3	2		2		2	252	0	1
8	9	432	2	2		2		2	91,2	0	1
5	5,5	88	2	9	0,41	9	0,47	2	124	0	1
8	8	180	2	2		2		2	27	0	0
4	4,8	30	2	9		9		2	126	1	0
1,8	2	2,5	2	2	1,4	9	0,9	2	38,6	1	0
6,1	5,2	68,2	3	2	3,84	2	8,68	2	120	0	0
3,1	3,6	22,3	2	2	1,8	2	5	2	27,5	0	0
2,1	2,2	7,6	2	9	0,43	9	0,85			1	0
2	4,2	15,12	3	2		2				1	0
			2	2	2,58	2	3,61	2	68,7	0	0

LKSCHW	FIEBER	REDAZ	GEWICHT	SCHMERZ	ATAXIE	DURCHF	QUKOMP	QUINK	HORNER	HYPERTONIE	BRILLENHÄM
0	0	0	0	0	0	0	0	0	0	0	0
0	0	0	0	0	0	0	0	0	0	0	0
0	0	0	0	1	0	0	0	0	0	0	0
0	0	0	0	0	0	0	0	0	0	0	0
0	0	0	0	0	0	0	0	0	0	0	0
0	0	0	0	0	0	0	0	0	0	0	0
0	1	0	0	1	0	0	0	0	0	0	0
0	0	0	0	0	0	0	0	0	0	0	0
0	0	0	0	0	0	0	0	0	0	0	0
0	0	0	0	1	0	0	0	0	0	0	0
0	0	0	0	0	0	0	0	0	0	0	0
0	0	0	0	0	0	0	0	0	0	0	0
0	0	0	0	0	0	0	0	0	0	0	0
0	0	0	0	0	0	0	0	0	0	0	0
0	0	0	0	0	0	0	0	0	0	0	0
0	0	0	0	1	0	0	0	0	0	0	0
0	0	0	0	0	0	0	0	0	0	0	0
0	0	0	0	0	0	0	0	0	0	0	0
0	0	0	0	0	0	0	0	0	0	0	0
0	0	0	0	0	0	1	0	0	0	0	0
0	0	0	0	0	0	0	0	0	0	0	0
0	0	0	0	0	0	0	0	0	0	0	0
0	0	0	0	0	0	0	0	0	0	0	0
0	0	0	0	0	0	0	0	0	0	0	0
0	0	0	0	0	0	0	0	0	0	0	0
0	0	0	0	1	0	0	0	0	0	0	0
0	0	0	0	0	0	0	0	0	0	0	0
0	0	0	0	0	0	0	0	0	0	0	0
0	0	0	0	1	0	0	0	0	0	0	0
0	0	0	0	0	0	0	0	0	0	0	0
0	0	0	0	0	0	0	0	0	0	0	0
0	0	0	0	0	0	0	0	0	0	0	0
0	0	0	0	1	0	0	0	0	0	0	0
0	0	0	0	1	0	0	0	0	0	0	0
0	0	0	1	1	0	0	0	0	0	0	0
0	0	0	0	1	0	0	0	0	0	0	0
0	1	0	0	1	0	0	0	0	0	0	0
0	0	0	0	0	0	0	0	0	0	0	0
0	0	0	0	0	0	0	0	0	0	0	0
0	0	0	0	1	0	0	0	0	0	0	0
0	1	0	0	0	0	0	0	0	0	0	0
1	0	0	0	0	0	0	0	0	0	0	0

0	0	0	0	0	0	0	0	0	0	0	0
0	0	0	0	0	0	0	0	0	0	0	0
0	0	0	0	0	0	0	0	0	0	0	0
0	0	0	0	0	0	0	0	0	0	0	0
0	0	0	0	1	0	0	0	0	0	0	0
0	1	0	0	1	0	0	0	0	0	0	0
0	0	0	0	1	0	0	0	0	0	0	0
0	0	0	0	0	0	0	0	0	0	0	0
0	0	0	0	1	0	0	0	0	0	0	0
0	1	0	0	0	0	0	0	0	0	0	0
0	0	0	0	0	0	0	0	0	0	0	0
0	0	0	0	1	0	0	0	0	0	0	0
0	0	0	0	0	0	0	0	0	0	0	0
0	0	0	0	0	0	1	0	0	0	0	0
0	0	0	0	0	0	0	0	0	0	0	0
0	0	0	0	0	0	0	0	0	0	0	0
0	0	0	1	0	0	0	0	0	0	0	0
0	0	0	0	0	0	0	0	0	0	0	0
0	1	1	0	1	0	0	0	0	0	0	0
0	0	0	0	0	0	0	0	0	0	0	0
0	0	0	0	0	0	0	0	0	0	0	0
0	0	0	0	1	0	0	0	0	0	0	0
0	0	0	0	0	0	0	0	0	0	0	0
0	0	0	0	0	0	0	0	0	0	0	0
0	0	0	1	1	0	0	0	0	0	0	0
0	0	0	0	0	0	0	0	0	0	0	0
0	0	0	0	0	0	0	0	0	0	0	0
0	0	0	0	1	0	0	0	0	0	0	0
0	0	0	0	1	0	0	0	0	0	0	0
0	0	0	0	0	0	0	0	0	0	0	0

0	0	0	0	0	1	0	0	0	0	0	0
0	1	0	0	1	0	0	0	0	0	0	0
0	0	0	0	1	0	0	0	0	0	0	0
0	0	0	0	0	0	0	0	0	0	0	0
0	1	0	0	0	1	0	0	0	0	0	0
0	0	0	0	0	0	0	0	0	0	0	0
0	0	0	0	0	0	0	0	0	0	0	0
0	0	0	0	0	0	0	0	0	0	0	0
0	0	0	0	0	0	0	0	0	0	0	0
0	0	0	0	1	0	0	0	0	0	0	0
0	1	0	0	0	0	0	0	0	0	0	0
0	0	0	0	1	0	0	0	0	0	0	0

0	0	1	1	0	0	0	0	0	0	0	0
0	0	0	0	0	0	0	0	0	0	0	0
0	0	0	0	0	0	0	0	0	0	0	0
0	0	0	0	1	0	0	0	0	0	0	0
0	1	0	0	1	0	0	0	0	0	0	0
0	0	0	0	0	0	0	0	0	1	0	0
0	0	0	0	1	0	0	0	0	0	0	0
0	0	0	0	1	0	0	0	0	0	0	0
0	0	1	0	1	0	0	0	0	0	0	0
0	0	0	0	1	0	0	0	0	0	0	0
0	0	0	0	0	0	0	0	0	0	0	0
0	0	0	0	0	0	0	0	0	0	0	0
0	0	1	0	1	0	0	0	0	0	0	0
0	0	0	0	1	0	0	0	0	0	0	0
0	0	0	0	0	0	0	0	0	0	1	0
1	0	0	0	0	0	0	0	0	0	0	0
0	0	0	0	0	0	0	0	0	0	0	0
0	0	0	0	1	0	0	0	0	0	0	0
0	1	0	0	0	0	0	0	0	0	0	0
0	0	1	0	0	0	0	0	0	0	0	0
0	0	0	0	0	0	0	0	0	0	0	0
0	0	0	0	1	0	0	0	0	0	0	0
0	0	0	0	1	0	0	0	0	0	0	0
0	0	0	0	0	0	0	0	0	0	0	0
0	0	0	0	0	0	0	0	0	0	0	0
0	0	0	0	0	0	0	0	0	0	0	0
0	0	0	0	0	0	0	0	0	0	0	0
0	0	1	0	1	0	0	0	0	0	0	0
0	0	0	0	1	1	0	0	0	0	0	0
0	0	0	0	0	0	0	0	0	0	0	0
0	1	0	0	0	0	0	0	0	0	0	0
0	0	0	0	0	0	0	0	0	0	0	0
0	0	0	0	1	0	0	0	0	0	0	0
0	0	0	0	1	0	0	0	0	0	0	0
0	1	0	0	0	0	0	0	0	0	0	0
0	0	0	0	1	0	0	0	0	0	0	0
0	0	0	0	0	0	0	0	0	0	0	0
0	0	0	0	0	0	0	0	0	0	0	0
0	0	0	0	0	0	0	0	0	0	0	0
0	1	1	0	1	0	0	0	0	0	0	0
0	0	0	0	0	1	0	0	0	0	0	0
0	0	1	0	0	0	1	0	0	0	0	0
0	1	0	0	0	0	0	0	0	0	0	0

0	0	0	0	0	0	0	0	0	0	0	0
0	0	0	0	0	0	0	0	0	0	0	0
0	0	0	0	0	0	0	0	0	0	0	0
0	0	0	0	1	0	0	0	0	0	0	0
0	0	0	0	0	0	0	0	0	0	0	0
0	0	0	0	0	0	0	0	0	0	0	0
0	0	0	0	1	0	0	0	0	0	0	0
0	0	0	1	1	0	0	0	0	0	0	0
0	0	0	0	0	0	0	0	0	0	0	0
0	0	0	0	0	0	0	0	0	0	0	0
0	0	0	0	0	0	0	0	0	0	0	0
0	0	0	0	0	0	0	0	0	0	0	0
0	0	0	0	0	0	0	0	0	0	0	0
0	0	0	0	1	0	0	0	0	0	0	0
0	0	0	0	0	0	0	0	0	0	0	0
0	0	0	0	0	0	0	0	0	0	0	0
0	0	0	0	0	0	0	0	0	0	0	0
0	0	0	0	1	0	0	0	0	0	0	0
0	0	0	0	0	0	0	0	0	1	0	0
0	0	0	0	0	0	0	0	0	0	0	0
0	1	1	1	0	0	0	0	0	0	0	0
0	1	0	0	1	0	0	0	0	0	0	0
0	0	0	0	0	0	0	0	0	0	0	0
0	0	0	0	1	0	0	0	0	0	0	0
0	0	0	0	0	0	0	0	0	0	0	0
0	0	1	0	1	0	0	0	0	0	0	0
0	0	0	0	0	0	0	0	0	0	0	0
0	0	0	0	0	0	0	0	0	0	0	0
0	0	0	0	1	0	0	0	0	0	0	0
0	1	0	0	1	0	0	0	0	0	0	0
0	0	0	0	1	0	0	0	0	0	0	0
0	0	0	0	0	0	0	0	0	0	0	0
0	0	0	0	0	0	1	0	0	0	0	0
0	1	0	0	1	0	0	0	0	0	0	0
0	0	1	0	0	0	0	0	0	0	0	0
0	0	0	0	0	1	0	0	0	0	0	0
0	0	0	0	0	0	0	0	0	0	0	0
0	0	0	0	0	0	0	0	0	0	0	0
0	0	0	0	0	0	0	0	0	0	0	0
0	0	0	0	0	0	0	0	0	0	0	0
0	0	0	0	1	0	0	0	0	0	0	0
0	0	0	0	0	0	0	0	0	0	0	0
0	0	0	0	0	0	0	0	0	0	0	0

0	0	0	0	0	0	0	0	0	0	0	0
0	0	0	0	1	0	0	0	0	0	0	0
0	0	0	0	0	0	0	0	0	0	0	0
0	0	0	0	0	0	0	0	0	0	0	0
0	0	0	0	0	0	0	0	1	0	0	0
0	0	0	0	0	0	0	0	0	0	0	0
0	0	0	0	0	0	0	0	0	0	0	0
0	0	0	0	0	0	0	0	0	0	0	0
0	1	1	0	0	0	0	0	0	0	0	0
0	0	1	0	0	0	0	0	0	0	0	0
0	0	0	0	1	0	0	0	0	0	0	0
0	0	0	0	0	0	0	0	0	0	0	0
0	0	0	0	0	0	0	0	0	0	0	0
0	0	0	0	0	0	0	0	0	0	0	0
0	0	0	0	0	0	0	0	0	0	0	0
1	0	0	0	0	0	0	0	0	0	0	0
0	0	0	0	0	0	1	0	0	0	0	0
0	0	0	0	0	0	0	0	0	0	0	0
0	0	0	0	0	0	0	0	0	0	0	0
0	0	0	0	1	0	0	0	0	0	0	0
0	0	0	0	0	0	0	0	0	0	0	0
0	1	0	0	0	0	0	0	0	0	0	0
0	0	0	0	0	0	0	0	0	0	0	0
0	0	0	1	1	0	0	0	0	0	0	0
0	0	0	0	0	0	0	0	0	0	0	0
0	0	0	0	0	0	0	0	0	1	0	0
0	0	0	0	0	0	0	0	0	0	0	0
0	0	0	0	0	0	0	0	0	0	0	0
1	0	0	0	0	0	0	0	0	1	0	0
0	0	0	0	0	0	1	0	0	0	0	0
0	0	0	0	0	0	0	0	0	0	0	0
0	0	0	0	0	0	0	0	0	0	0	0
0	0	0	0	0	0	0	0	0	0	0	0
0	0	0	0	0	0	0	0	0	0	0	0
0	0	0	0	0	0	0	0	0	0	0	0
0	0	0	0	0	0	0	0	0	0	0	0
0	0	0	0	0	0	0	0	0	0	0	0
0	0	0	0	1	0	0	0	0	0	0	0
0	0	0	1	0	0	0	0	0	0	0	0
0	0	0	0	0	0	0	0	0	0	0	0
0	0	0	0	0	0	0	0	0	0	0	0
0	0	0	0	1	0	0	0	0	0	0	0
0	0	0	0	0	0	0	0	0	0	0	0

0	0	0	0	0	0	0	0	0	0	0	0
0	0	0	0	0	0	0	0	0	0	0	0
0	1	1	0	1	0	0	0	0	0	0	0
0	0	0	0	1	0	0	0	0	0	0	0
0	0	0	0	0	0	0	0	0	0	0	0
0	0	0	0	0	0	0	0	0	0	0	0
0	0	0	0	0	1	0	0	0	0	0	0
0	0	0	0	0	0	0	1	0	0	0	0
0	0	0	0	0	0	0	0	0	0	0	0
0	0	1	1	1	0	0	0	0	0	0	0
0	0	0	0	0	1	0	0	0	0	0	0
0	1	0	0	0	0	0	0	0	0	0	0
0	0	0	0	0	0	0	0	0	0	0	0
0	0	0	0	0	0	0	0	0	0	0	0
0	0	0	0	0	0	0	0	0	0	0	0
0	0	0	0	0	0	0	0	0	0	0	0
0	0	0	0	0	0	0	0	0	1	0	0
0	0	0	0	0	0	0	0	0	0	0	0
0	0	0	0	0	0	0	0	0	0	0	0
0	0	0	0	0	0	0	0	0	0	0	0
0	0	0	0	1	0	0	0	0	0	0	0
0	0	0	0	0	0	0	0	0	0	0	0
0	0	0	0	0	0	0	0	0	0	0	0
0	0	0	0	0	0	0	0	1	0	0	0
0	0	0	0	0	0	0	0	1	0	0	0
0	0	0	0	0	0	0	0	0	0	0	0
0	0	0	0	0	0	0	0	0	0	0	0
0	0	0	0	0	0	0	0	0	0	0	0
0	0	0	0	0	0	0	0	0	0	0	0
0	0	1	1	1	0	0	0	1	0	0	0
0	0	0	0	0	0	0	0	0	0	0	0
0	0	0	0	1	0	0	0	0	0	0	0
0	0	0	0	0	0	0	0	1	0	0	0
0	1	0	0	0	0	1	0	0	0	0	0
0	1	0	0	1	0	0	0	0	0	0	0
0	0	0	0	0	1	0	0	0	0	0	0
0	0	1	0	0	1	0	0	0	0	0	0
0	0	1	0	0	1	0	0	0	0	0	0
0	0	1	0	0	0	0	0	0	0	0	0
0	0	0	0	1	0	0	0	0	0	0	0
0	0	1	0	0	0	0	1	0	0	0	0
0	0	0	0	0	0	0	0	0	1	0	0
0	0	0	0	0	0	0	0	0	1	0	0

0	0	0	0	0	0	0	0	0	0	0	0
0	0	0	0	0	0	0	0	0	0	0	0
0	0	0	0	0	1	0	0	0	0	0	0
0	0	0	1	1	0	0	0	0	0	0	0
0	1	1	1	0	0	0	0	0	0	0	0
0	0	0	0	0	0	0	0	0	0	0	0
0	0	0	0	0	0	0	0	0	0	0	0
0	0	0	0	0	0	0	0	0	0	0	0
0	0	0	0	0	0	0	0	0	0	0	0
0	0	1	0	0	1	0	0	0	0	0	0
0	0	0	0	0	0	0	0	0	0	0	0
0	0	0	0	0	0	0	0	0	0	0	0
0	0	0	0	0	0	0	0	0	0	0	0
0	0	0	0	0	0	0	0	0	0	0	0
0	0	0	0	0	0	0	0	0	0	0	0
0	0	0	0	0	0	0	0	0	0	0	0
0	0	0	0	0	0	0	0	0	0	0	0
0	0	0	0	0	0	0	0	0	0	0	0
0	0	0	0	0	0	0	0	0	0	0	0
0	0	0	0	0	0	0	0	0	0	0	0
0	0	0	0	0	0	0	0	0	0	0	0
0	0	0	0	0	0	0	0	0	0	0	0
0	0	1	0	1	0	0	0	0	0	0	0
0	0	0	0	0	0	0	0	0	0	0	0
0	0	0	0	0	0	0	0	0	0	0	0
0	0	0	0	0	0	0	0	0	0	0	0
0	0	0	0	0	0	0	0	0	0	0	0
0	0	0	0	0	0	0	0	0	0	0	0
0	0	0	0	0	0	0	0	0	0	0	0
0	0	0	0	0	0	0	0	1	0	0	0
0	0	1	0	0	0	0	0	0	0	0	0
0	0	0	0	0	0	0	0	0	0	0	0
0	0	0	0	0	0	0	0	0	0	0	0
0	0	0	0	0	0	0	0	0	0	0	0
0	0	0	0	0	0	0	0	0	0	0	0
0	0	0	0	0	0	0	0	0	0	0	0
0	0	0	0	0	0	0	0	0	0	0	0
0	0	0	0	0	0	0	0	0	0	0	0
0	0	0	0	0	0	0	0	1	0	0	0
0	0	0	0	0	0	0	0	0	1	0	0
0	0	0	0	0	0	0	0	0	0	0	0
0	1	1	0	1	0	0	0	0	0	0	0
0	0	0	0	0	0	0	0	0	0	0	0

0	0	1	1	1	0	0	0	1	0	0	0
0	0	0	0	0	0	0	0	0	0	0	0
0	0	1	0	0	0	0	0	0	0	0	0
0	0	0	0	0	0	0	0	0	0	0	0
0	1	1	1	1	0	0	0	1	0	0	0
0	0	0	0	0	0	0	0	0	0	0	0
0	1	1	0	1	0	0	0	0	0	0	0
0	0	0	0	0	0	0	0	0	0	0	0
0	0	0	0	0	0	0	0	0	0	0	0
0	0	0	0	0	0	0	0	0	0	0	0
0	0	0	0	0	0	0	0	0	0	0	0
0	0	0	0	0	0	0	0	0	0	0	0
0	0	0	0	0	0	0	0	0	0	0	0
0	0	0	0	0	0	0	0	0	0	0	0
0	0	0	0	0	0	0	0	0	0	0	0
0	1	1	0	1	0	1	0	0	0	0	0
0	0	0	0	0	0	0	0	0	0	0	0
0	0	0	0	0	0	0	0	0	0	0	0
0	0	0	0	0	0	0	0	0	0	0	0
0	0	0	0	0	0	0	0	0	0	0	0
								1			0
0	0	0	0	1	0	0	0	0	0	0	0
0	0	0	0	0	0	0	0	0	0	0	0
0	0	0	0	0	0	0	0	0	1	0	0
0	0	0	0	0	0	0	0	1	0	0	0
0	0	0	0	0	0	0	0	0	0	0	0
0	0	0	0	0	0	0	0	0	0	0	0
0	0	1	0	1	0	0	0	0	0	0	0
0	0	0	0	0	0	0	0	0	0	0	0
0	0	1	0	1	0	0	0	0	0	0	0
0	0	0	0	0	0	0	0	0	0	0	0
0	0	0	0	0	0	1	0	0	0	0	0
0	0	0	0	0	0	0	0	0	0	0	0
0	0	0	0	0	1	0	0	0	0	0	0
0	0	0	0	0	0	0	0	0	0	0	0
0	0	0	0	0	0	0	0	0	0	0	0
0	0	0	0	0	0	0	0	0	0	0	0
0	0	1	1	1	0	0	0	0	0	0	0
0	0	0	0	0	0	0	0	0	0	0	0
0	0	0	0	0	0	0	0	0	0	0	0
0	0	0	0	0	0	0	0	0	0	0	0
0	0	0	0	0	0	0	0	0	0	0	0
0	0	0	0	0	0	0	0	0	0	0	0

0	1	1	0	1	0	0	0	0	0	0	0
0	0	0	0	0	0	0	0	0	0	0	0
0	1	1	0	0	0	0	0	1	0	0	0
0	0	0	0	0	0	0	0	0	0	0	0
0	0	0	0	0	0	0	0	0	0	0	0
0	0	0	0	0	0	0	0	0	0	0	0
0	0	0	0	0	0	0	0	0	0	0	0
0	0	0	0	1	0	0	0	0	0	1	0
0	0	0	0	0	0	0	0	0	0	0	0
0	0	1	0	0	0	0	0	1	0	0	0
0	0	0	0	0	0	0	0	0	0	0	0
0	0	0	1	0	0	0	0	0	0	0	0
0	0	0	0	0	0	0	0	0	0	0	0
0	0	1	0	0	0	0	0	0	0	0	0
0	0	1	0	0	0	1	0	0	0	1	0
0	0	0	0	0	0	0	0	0	0	0	0
0	0	0	0	0	1	0	0	0	0	0	0
0	0	0	0	0	0	0	0	0	0	0	0
0	1	0	0	0	0	0	0	0	0	0	0
0	0	0	0	0	0	0	0	0	0	0	0
0	0	0	0	0	0	0	0	0	0	0	0
0	0	0	0	0	0	0	0	0	0	0	0
0	0	0	0	0	0	0	0	0	0	0	0
0	0	0	0	0	1	0	0	0	0	0	0
0	0	0	0	0	0	0	0	0	0	0	0

0	0	0	0	0	0	0	0	0	0	0	0
0	0	0	0	0	0	0	0	0	0	0	0
0	0	0	0	0	0	0	0	0	0	0	0
0	0	1	0	0	0	0	0	0	0	0	0
0	0	0	0	0	0	0	0	0	0	0	0
0	1	1	0	0	0	0	0	0	0	0	0
0	0	0	0	0	0	0	0	0	0	0	0
0	0	0	0	0	0	0	0	0	0	0	0
0	0	0	0	0	0	0	0	0	1	0	0
0	0	0	0	1	0	0	0	0	0	0	0
0	0	0	0	0	0	0	0	0	0	0	0
0	0	0	0	0	0	0	0	0	0	0	0
0	0	1	0	0	0	1	0	0	0	0	0
0	0	0	0	0	0	0	0	0	0	0	0
0	0	0	0	0	0	0	0	0	0	0	0
0	0	0	1	1	0	0	0	0	0	0	0
0	0	0	0	0	0	0	0	0	0	0	0

0	1	0	0	0	0	1	0	0	0	0	0
0	0	0	0	0	0	0	0	0	0	0	0
0	0	0	0	0	0	0	0	0	0	0	0
0	0	0	0	0	0	0	0	0	0	0	0
0	0	0	0	0	0	0	0	0	0	0	0
0	0	0	0	1	0	0	0	0	0	1	0
0	0	1	1	1	0	0	0	0	0	0	0
0	0	0	0	0	0	0	0	1	0	0	0
0	0	0	0	1	0	0	0	0	0	0	0
0	0	0	0	0	0	0	0	0	0	0	0
0	0	0	0	0	0	0	0	0	0	0	0
0	1	1	0	0	0	0	0	0	0	0	0
0	1	1	0	0	0	0	0	1	0	0	0
0	0	0	0	0	0	0	0	0	0	0	0
0	0	0	0	0	0	0	0	0	0	0	0
0	0	0	0	0	0	0	0	0	0	0	0
0	0	0	0	0	0	0	0	0	0	0	0
0	0	0	1	0	1	0	0	0	0	0	0
1	1	1	0	0	0	0	0	0	0	0	0
0	0	0	0	0	0	0	0	0	0	0	0
0	0	0	0	0	0	0	0	0	0	0	0
0	0	0	0	0	0	0	0	0	0	0	0
0	0	0	0	0	0	0	0	0	0	0	0
0	0	0	0	0	0	0	0	0	0	0	0
0	0	0	0	0	0	0	0	0	0	0	0
0	0	0	0	0	0	0	0	0	0	0	0
0	0	0	0	0	0	0	0	0	0	0	0
0	0	0	0	0	0	0	0	0	0	0	0
0	0	0	0	0	0	0	0	0	0	0	0
0	0	1	0	0	0	0	0	0	1	0	0
0	0	0	0	0	0	0	0	0	0	0	0
0	0	0	0	0	0	0	0	1	0	0	0
0	0	1	0	0	1	0	0	0	0	0	0
0	0	0	0	0	0	0	0	0	0	0	0
0	0	0	0	0	0	0	0	0	0	0	0
0	0	0	0	0	0	0	0	0	0	0	0
0	0	0	0	0	0	0	0	0	0	0	0
0	0	0	0	0	0	0	0	0	0	0	0
0	1	1	0	0	0	0	0	0	0	0	0
0	0	1	1	0	0	0	0	0	0	1	0
0	0	0	0	0	0	0	0	0	0	0	0
0	0	0	1	0	0	0	0	0	0	0	0
0	0	0	0	0	0	0	0	0	0	0	0
1	0	0	0	0	0	0	0	0	0	0	0

1	0	0	0	0	0	0	0	0	0	0	0
0	0	0	0	0	0	0	0	0	0	0	0
0	0	1	0	0	0	0	0	1	0	0	0
0	0	0	0	0	0	0	0	0	0	0	0
0	0	0	0	0	0	0	0	0	0	0	0
0	0	0	0	0	0	0	0	0	0	0	0
0	0	1	1	1	0	0	0	0	0	0	0
0	1	0	0	1	0	0	0	0	0	0	0
0	0	0	0	0	0	0	0	0	0	0	0
0	0	0	0	0	0	0	0	0	0	0	0
0	0	0	1	1	0	0	0	0	0	0	0
0	0	1	0	0	0	0	0	0	0	0	0
0	0	0	0	0	0	0	0	0	0	0	0
0	1	1	0	1	0	0	0	0	0	0	0
0	0	0	0	0	0	0	0	0	0	0	0
0	0	0	0	0	0	0	0	0	1	0	0
0	0	0	0	0	0	0	0	0	0	0	0
0	0	0	0	0	0	0	1	0	0	0	0
0	0	0	0	0	0	0	0	0	0	0	0
0	0	1	0	1	0	0	0	1	0	0	0
0	1	0	0	0	0	0	0	0	0	0	0
0	0	0	0	0	0	0	0	0	0	0	0
0	0	0	0	0	0	0	0	0	0	0	0
0	0	0	0	0	0	0	0	0	0	0	0
1	0	0	0	0	0	0	0	0	0	0	0
0	0	0	0	1	0	0	0	0	0	0	0
0	1	1	0	0	0	0	0	0	0	0	0
0	0	0	0	0	0	0	0	0	0	0	0
0	0	0	0	0	0	0	0	0	0	0	0
0	0	0	0	0	0	0	0	0	0	0	0
0	0	0	0	0	0	0	0	1	0	0	0
0	0	0	0	1	0	0	0	0	0	0	0
0	0	0	1	0	0	0	0	0	0	0	0
0	0	0	0	0	0	0	0	0	0	0	0
0	0	0	0	0	0	0	0	0	0	0	0
0	1	0	1	1	0	0	0	0	0	0	0
0	0	0	0	0	0	0	0	0	0	0	0
0	0	0	0	0	0	0	0	0	0	0	0
0	0	0	0	0	0	0	0	0	0	0	0
0	0	0	0	0	0	0	0	0	0	0	0
0	0	0	0	0	0	0	0	0	0	0	0
0	0	0	0	0	0	0	0	0	0	0	0
0	0	0	0	0	0	0	0	0	0	0	0

0	0	0	0	0	0	0	0	0	0	0	0
0	0	0	0	0	0	0	0	0	0	0	0
0	1	0	0	0	0	0	0	0	0	0	0
0	1	1	0	0	0	0	0	0	0	0	0
0	0	0	0	0	0	0	0	0	0	0	0
0	0	0	0	0	0	0	0	0	0	0	0
0	0	0	0	0	0	0	0	0	0	0	0
0	0	0	0	0	0	0	0	0	0	0	0
0	0	0	0	0	0	0	0	0	0	0	0
0	0	0	0	0	0	0	0	0	0	0	0
0	0	0	0	0	0	0	0	0	0	0	0
0	0	0	0	0	0	0	0	0	0	0	0
0	0	0	0	0	0	0	0	1	0	0	0
0	0	0	0	0	0	0	0	0	0	0	0
0	0	0	0	0	0	0	0	0	0	0	0
0	0	0	0	0	1	0	0	0	0	0	0
0	1	1	0	0	0	1	0	0	0	0	0
0	0	0	0	0	0	0	0	0	0	0	0
0	1	1	1	0	0	1	0	0	0	0	0
0	0	0	0	0	0	0	0	0	0	0	0
0	0	0	0	0	0	0	0	0	1	0	0
0	0	0	0	0	0	0	0	0	0	0	0
0	1	0	0	1	0	0	0	0	0	0	0
0	0	0	0	0	0	0	0	0	0	0	0
0	0	0	0	0	0	0	0	0	0	0	0
0	0	0	0	0	0	0	0	0	0	0	0
0	0	1	0	0	0	0	0	0	0	0	0
0	0	0	0	0	0	0	0	0	0	1	0
1	0	0	0	0	0	0	0	0	0	0	0
0	0	0	0	0	0	0	0	0	0	0	0
0	0	0	0	0	0	0	0	0	0	0	0
0	0	1	1	0	0	0	0	0	0	1	0
0	0	0	0	0	0	0	0	0	0	0	0
0	0	0	0	0	0	0	0	0	0	0	0
0	0	0	0	0	0	0	0	0	0	0	0
0	1	1	0	1	0	0	0	0	0	0	0
0	0	0	0	0	0	0	0	0	0	0	0
0	0	1	1	1	0	1	0	0	0	0	0
0	0	1	0	1	0	0	0	0	0	0	0
0	0	0	0	0	0	0	0	0	0	0	0
0	0	0	0	0	0	0	0	0	0	0	0
0	0	0	0	0	0	0	0	0	0	0	0
0	0	0	0	0	0	0	0	0	0	0	0

0	0	0	0	0	0	0	0	0	0	0	0
0	0	0	0	0	0	0	0	0	0	0	0
0	0	0	0	0	1	0	0	0	0	0	0
0	0	0	0	0	0	0	0	0	0	0	0
0	0	0	0	1	0	0	0	0	0	0	0
0	0	0	0	0	0	0	0	0	0	0	0
0	0	0	0	0	0	0	0	0	0	0	0
0	0	0	0	0	0	0	0	0	0	0	0
0	0	0	0	0	0	0	0	0	1	0	0
0	0	0	0	0	0	0	0	0	0	0	0
0	1	1	0	0	0	0	0	0	0	0	0
0	0	0	0	0	0	0	0	0	0	0	0
0	0	0	0	0	0	0	0	0	0	0	0
0	0	0	0	0	1	0	0	0	0	0	0
0	0	0	0	0	0	0	0	0	0	0	0
0	0	1	0	0	0	0	0	0	0	0	0
0	0	0	0	0	0	0	0	0	0	0	0
0	0	0	0	0	0	0	0	0	0	0	0
0	0	0	0	0	1	0	0	0	0	0	0
0	0	0	0	0	0	0	0	0	0	0	0
0	0	0	0	0	0	0	0	0	0	0	0
0	0	0	0	0	0	0	0	0	0	0	0
0	0	0	0	0	0	0	0	0	1	0	0
0	0	0	1	0	0	0	0	0	0	0	0
0	1	1	0	0	0	0	0	0	0	0	0
0	1	1	1	1	0	0	0	0	0	0	0
0	0	0	0	0	0	0	0	0	0	0	0
0	0	1	0	0	0	0	0	0	0	0	0
0	0	0	0	0	0	0	0	0	0	0	0
0	0	0	0	0	0	0	0	0	0	0	0
0	0	0	0	0	1	0	0	0	0	0	0
0	0	0	0	0	1	0	0	0	0	0	0
0	1	0	1	0	0	0	0	0	0	0	0
0	1	1	0	0	0	0	0	0	0	1	0
0	0	0	0	0	0	0	0	0	0	0	0
0	0	0	0	0	0	0	0	0	0	0	0
0	0	0	0	0	0	0	0	0	0	0	0
0	1	0	0	0	0	0	0	0	0	0	0
0	0	0	1	1	0	0	0	0	0	0	0
0	0	0	0	0	0	0	0	0	0	0	0
0	0	0	0	0	0	0	0	0	0	0	0
0	0	0	0	0	0	0	0	0	0	0	0
0	0	0	0	0	0	0	0	1	0	0	0

0	0	0	0	0	0	0	0	0	0	0	0
0	0	0	0	1	0	0	0	0	0	0	0
0	0	0	0	0	0	0	0	0	0	0	0
0	0	0	0	0	0	0	0	0	0	0	0
0	0	0	0	0	0	0	0	0	0	0	0
0	0	0	0	0	0	0	0	0	0	1	0
0	0	0	0	0	0	0	0	0	0	0	0
0	0	1	1	0	0	0	0	0	0	1	0
0	0	0	0	0	0	0	0	0	0	0	0
0	0	0	0	0	0	0	0	0	0	0	0
0	0	0	0	0	0	0	0	0	0	0	0
0	0	1	1	0	0	0	0	0	0	0	0
0	0	0	0	0	0	0	0	0	0	0	0
0	0	0	0	0	0	0	0	0	0	0	0
0	0	0	0	0	0	0	0	0	0	0	0
0	1	0	0	0	0	0	0	0	0	0	0
0	0	0	0	0	0	0	0	0	0	0	0
0	0	1	0	0	0	0	0	0	1	0	0
0	0	0	0	0	0	0	0	0	0	0	0
0	0	0	0	0	0	0	0	0	0	0	0
0	0	0	0	0	0	0	0	0	0	0	0
0	0	0	0	0	0	0	0	0	0	0	0
0	0	0	0	0	0	0	0	0	0	0	0
		1		1							0
0	0	1	1	0	0	0	0	0	0	0	0
0	1	0	0	1	0	0	0	0	0	0	0
0	0	0	0	1	0	0	0	0	0	0	0
0	0	0	0	0	0	0	0	0	0	0	0
0	0	0	0	0	0	0	0	0	0	0	0
0	0	0	0	0	0	0	0	0	0	0	0
0	0	0	0	0	0	0	0	0	0	0	0
0	0	0	0	0	0	0	0	0	0	0	0
0	0	0	0	0	1	0	0	0	0	0	0
0	0	0	0	0	0	0	0	1	0	0	0
0	0	0	0	0	0	0	0	0	0	0	0
0	0	0	0	1	0	0	0	1	0	0	0
0	1	1	0	0	0	0	0	0	0	0	0
0	0	0	0	0	0	0	0	0	0	0	0
0	0	0	0	0	0	0	0	0	0	0	0
0	0	0	0	0	0	0	0	0	0	0	0
0	0	0	0	0	0	0	0	0	0	0	0
0	0	0	0	0	0	0	0	0	0	0	0
0	0	0	0	1	0	0	0	1	0	0	0

0	0	1	0	1	0	0	0	0	0	0	0
0	0	0	0	0	0	0	0	0	0	0	0
0	0	0	0	0	0	0	0	0	0	0	0
0	1	1	0	0	0	0	0	0	0	0	0
0	0	0	0	0	1	0	0	0	0	0	0
0	0	0	0	0	0	0	0	0	0	0	0
0	0	0	0	1	0	0	0	0	0	0	0
0	0	0	0	0	0	0	0	0	0	0	0
0	0	0	0	0	0	0	0	0	0	0	0
0	0	0	0	0	0	0	0	0	0	0	0
0	0	0	0	0	0	0	0	0	0	0	0
0	0	0	0	0	0	0	0	0	0	0	0
0	0	0	0	0	0	0	0	0	0	0	0
0	0	0	0	0	0	0	0	0	0	0	0
0	0	0	0	0	0	0	0	0	0	0	0
0	0	0	0	0	0	0	0	0	0	0	0
0	0	0	0	0	0	0	0	0	0	0	0
0	0	0	0	1	0	0	0	0	0	0	0
0	0	0	0	0	0	0	0	0	0	0	0
0	0	0	0	0	0	0	0	0	0	0	0
0	0	0	0	0	0	0	0	0	0	0	0
0	0	0	0	0	0	0	0	0	0	0	0
0	0	0	0	0	0	0	0	0	0	0	0
0	0	0	0	0	0	0	0	0	0	0	0
0	0	0	0	0	0	0	0	0	0	0	0
0	0	0	0	0	0	0	0	1	0	0	0
0	0	0	0	0	0	0	0	0	0	0	0
0	0	1	0	1	0	0	0	0	0	0	0
0	0	0	0	0	0	0	0	0	0	0	0
0	1	1	1	0	0	0	0	0	0	1	0
0	1	0	0	1	0	0	0	0	0	0	0
0	0	0	0	0	0	0	0	0	0	0	0
0	0	0	1	0	0	0	0	0	0	0	0
0	0	0	0	0	0	0	0	0	0	0	0
0	0	0	0	0	0	0	0	0	0	0	0
0	0	0	0	0	0	0	0	0	0	0	0
0	0	0	0	0	0	0	1	1	0	0	0
0	0	0	0	0	0	0	0	0	0	0	0
0	0	1	0	1	0	0	0	0	0	0	0
0	0	0	1	1	0	0	0	0	0	0	0
0	0	0	0	0	0	0	0	0	0	0	0
0	0	0	0	0	0	0	0	0	0	0	0
0	0	0	0	0	0	0	0	0	0	0	0

0	1	0	0	0	0	0	0	0	0	0	0
1	1	0	0	0	0	0	0	0	0	0	0
0	0	0	0	0	0	0	0	0	0	0	0
0	0	0	0	0	0	0	0	0	0	0	0
0	0	0	0	0	0	0	0	0	0	0	0
0	0	0	0	0	0	0	0	0	0	0	0
0	0	0	0	0	0	0	0	0	0	0	0
0	0	0	0	0	0	0	0	0	0	0	0
0	0	0	0	0	0	0	0	0	0	0	0
0	0	0	0	0	0	0	0	0	0	0	0
0	0	0	0	0	0	0	0	0	0	0	0
0	0	0	0	0	0	0	0	0	0	0	0
0	0	0	0	0	0	0	0	0	0	0	0
0	0	0	0	0	0	0	0	0	0	0	0
0	0	0	0	0	0	0	0	0	0	0	0
0	0	1	0	0	1	0	0	0	0	0	0
0	0	0	0	0	0	0	0	0	0	0	0
0	0	0	0	0	0	0	0	0	0	0	0
0	0	0	0	0	0	0	0	0	0	0	0
0	0	0	0	0	0	0	0	0	0	0	0
0	0	0	0	0	0	0	0	1	0	0	0
0	0	0	0	0	0	0	0	1	0	0	0
0	0	0	0	0	0	0	0	0	0	0	0
0	0	0	1	0	0	0	0	0	0	0	0
0	0	0	0	1	0	0	0	0	0	0	0
0	0	0	0	0	0	0	0	0	1	0	0
0	0	0	0	0	0	0	0	0	0	0	0
0	1	0	1	1	0	0	0	0	0	0	0
0	1	1	1	0	0	0	0	0	0	1	0
0	0	0	0	0	0	0	0	0	0	0	0
0	0	0	0	0	0	0	0	0	0	0	0
0	0	0	0	0	0	0	0	0	1	0	0
0	0	0	0	0	0	0	0	0	0	0	0
0	1	1	0	1	0	0	0	0	0	0	0
0	0	0	0	0	0	0	0	0	0	0	0
0	0	0	0	0	0	0	0	0	0	0	0
0	0	0	0	0	0	0	0	0	0	0	0
0	0	0	0	1	0	0	0	1	0	0	0
0	0	0	0	0	0	0	0	0	0	0	0
0	0	0	0	0	0	0	0	0	0	0	0
0	0	0	0	0	0	0	0	0	0	0	0
0	0	0	0	0	0	0	0	0	0	0	0
0	0	1	0	0	0	0	0	0	0	0	0

0	0	0	0	1	0	0	0	0	0	0	0
0	0	0	0	1	0	0	0	0	0	0	0
0	0	0	0	0	0	0	0	0	0	0	0
0	0	0	0	0	0	0	0	0	0	0	0
0	0	1	0	0	0	0	0	0	1	0	0
0	0	0	0	0	0	0	0	1	0	0	0
0	0	0	0	0	0	0	0	0	0	0	0
0	0	0	0	0	0	0	0	0	0	0	0
0	0	0	0	0	0	0	0	0	0	0	0
0	0	0	0	0	0	0	0	0	0	0	0
0	0	0	0	0	0	0	0	0	0	0	0
0	0	0	0	0	0	0	0	0	0	0	0
0	0	0	0	0	0	0	0	0	0	0	0
0	0	0	0	0	0	0	0	0	1	0	0
0	0	0	0	0	0	0	0	0	0	0	0
0	0	0	0	0	0	0	0	0	0	0	0
0	0	0	0	0	0	0	0	0	0	0	0
0	1	1	0	1	0	0	0	0	0	0	0
0	0	0	0	0	0	0	0	1	0	0	0
0	0	1	0	1	0	0	0	0	0	0	0
0	0	0	0	0	0	0	0	0	1	0	0
0	0	0	0	0	0	0	0	0	0	0	0
0	1	1	1	0	0	0	0	0	0	0	0
0	0	0	0	0	0	0	0	0	0	0	0
0	0	1	1	1	0	0	0	0	0	0	0
0	0	0	0	0	0	0	0	0	0	0	0
0	0	0	0	1	0	1	0	0	0	0	0
0	0	0	0	0	0	0	0	0	0	0	0
0	0	0	0	0	0	0	0	0	0	0	0
0	0	0	0	0	0	0	0	0	0	0	0
0	0	1	0	1	0	0	0	0	0	0	0
0	0	1	1	1	0	0	0	1	0	0	0
0	0	0	0	0	0	0	0	0	0	0	0
0	0	1	0	0	0	1	0	0	0	0	0
0	0	1	0	1	0	0	0	1	0	1	0
0	0	1	0	1	0	0	0	0	0	0	0
0	0	1	1	0	0	0	0	0	0	0	0
0	1	1	0	1	0	0	0	0	0	0	0
0	1	1	0	0	0	0	0	0	0	0	0
0	0	1	0	0	1	0	0	0	0	0	0
0	0	0	0	0	0	0	0	0	0	0	0
0	0	0	0	0	0	0	0	0	0	0	0
0	0	0	0	0	0	0	0	0	0	0	0

0	0	0	0	0	0	0	0	0	0	0	0
0	1	0	0	1	0	0	0	1	0	0	0
0	1	1	0	0	0	0	0	0	0	0	0
0	0	0	0	0	0	0	0	0	0	0	0
0	0	0	0	0	1	0	0	0	0	0	0
0	0	0	0	0	0	0	0	0	0	0	0
0	0	0	0	0	0	0	0	0	0	0	0
0	0	0	0	0	0	0	0	0	0	0	0
0	0	0	0	0	0	0	0	0	0	0	0
0	0	0	0	0	0	0	0	0	0	0	0
0	0	0	0	0	0	0	0	0	0	0	0
0	0	0	0	0	0	0	0	0	0	0	0
0	0	0	0	0	0	0	0	0	0	0	0
0	0	0	0	0	0	0	0	1	0	0	0
0	0	0	0	0	0	0	0	0	0	0	0
0	0	0	0	0	0	0	0	0	0	0	0
0	0	1	0	1	0	0	0	1	0	0	0
0	0	0	0	0	0	0	0	0	0	0	0
0	0	0	0	0	0	0	0	1	0	0	0
0	0	0	0	0	1	0	0	0	0	0	0
0	0	0	0	0	0	0	0	0	1	0	0
0	1	1	0	0	0	0	0	0	0	0	0
0	0	1	0	1	0	0	0	1	0	0	0
1	0	0	0	0	0	0	0	0	0	0	0
0	0	0	0	0	0	0	0	0	0	0	0
0	0	0	0	0	0	0	0	0	0	0	0
0	0	0	0	0	0	0	0	0	0	0	0
0	0	0	0	0	0	0	0	0	0	1	0
0	0	0	0	0	0	1	0	0	0	0	0
0	0	0	0	0	0	0	0	1	0	0	0
0	0	0	0	1	0	0	0	0	0	0	0
0	0	0	0	0	0	0	0	0	0	0	0
0	1	0	0	0	0	0	0	0	0	0	0
0	0	0	0	0	0	0	0	0	0	0	0
0	0	0	0	0	0	0	0	0	0	0	0
0	1	1	1	1	0	0	0	0	0	0	0
0	0	0	0	0	0	0	0	0	0	0	0
0	0	0	1	0	0	1	0	0	0	0	0
0	1	0	0	0	0	0	0	0	0	0	0
0	0	0	0	0	0	0	0	0	0	0	0
0	0	0	0	0	0	0	0	0	0	0	0
0	0	0	0	0	0	0	0	0	0	0	0
0	0	0	0	1	0	0	0	0	0	0	0

0	0	0	0	1	0	0	0	0	0	0	0
0	0	0	1	1	0	0	0	0	0	0	0
0	0	0	0	0	0	0	0	0	0	0	0
0	1	1	0	0	0	0	0	0	0	0	0
0	0	0	0	0	0	0	0	0	0	0	0
0	0	0	0	0	0	0	0	0	0	0	0
0	0	0	0	0	0	0	0	0	0	0	0
0	0	0	0	0	0	0	0	0	0	0	0
0	0	0	0	1	0	0	0	0	0	0	0
0	0	0	0	0	0	0	0	0	0	0	0
0	0	0	0	0	0	0	0	0	0	0	0
0	0	0	0	0	0	0	0	0	0	0	0
0	0	0	0	0	0	0	0	0	0	0	0
0	0	0	0	0	0	0	0	0	0	1	0
0	1	1	0	0	0	1	0	0	0	0	0
0	0	0	0	1	0	0	0	0	0	0	0
0	0	0	0	0	0	0	0	0	0	0	0
0	0	0	0	0	0	0	0	0	0	0	0
0	0	0	0	0	0	0	0	0	0	0	0
0	0	0	0	0	0	0	0	0	0	0	0
0	0	0	0	0	0	0	0	0	0	0	0
0	0	0	0	0	0	0	0	0	0	0	0
0	0	0	0	0	0	0	0	0	0	0	0
0	0	0	0	0	0	0	0	0	1	0	0
0	0	0	0	0	0	0	0	1	0	0	0
0	0	0	0	0	0	0	0	0	0	0	0
0	0	0	0	1	0	0	0	0	0	0	0
0	1	1	0	0	0	0	0	0	0	0	0
0	0	0	0	0	0	0	0	0	0	0	0
0	0	0	0	0	0	0	0	0	0	0	0
0	1	1	1	0	0	0	0	0	0	1	0
0	0	0	0	1	0	0	0	0	0	0	0
0	0	0	0	0	0	0	0	0	0	0	0
0	0	0	0	0	0	0	0	0	0	0	0
0	0	0	0	0	0	0	0	0	1	0	0

PATH_FRAK	ANZSYMP	VORER_SYNDR_NEIN
0	0	1
0	0	1
0	1	1
0	1	1
0	0	0
0	0	0
0	3	1
0	1	1
0	0	1
0	1	0
0	0	1
0	0	1
0	0	1
0	1	1
0	0	0
0	2	1
0	0	1
0	0	1
0	0	1
0	1	1
0	0	1
0	0	1
0	0	1
0	1	1
0	0	1
0	2	1
0	0	0
0	0	0
0	1	1
0	1	1
0	0	1
0	0	1
0	2	1
0	1	1
0	3	1
0	1	1
0	3	1
0	0	1
0	1	1
0	1	0
0	2	0
0	2	1

0	0	1
0	0	1
0	0	1
0	0	0
0	1	0
0	2	1
0	1	1
0	2	1
0	1	1
0	1	1
0	0	0
0	1	1
0	1	1
0	1	1
0	0	1
0	0	1
0	2	1
0	0	1
0	3	1
0	1	1
0	1	1
0	1	0
0	0	1
0	0	0
0	2	1
0	0	0
0	0	1
0	2	1
0	1	1
0	0	1
0	1	1
0	3	0
0	1	1
0	0	1
0	2	1
0	1	1
0	0	1
0	1	1
0	0	1
0	2	1
0	1	1
0	1	1

0	3	1
0	0	0
0	1	0
0	1	1
0	2	1
0	2	1
0	2	0
0	1	1
0	2	1
0	1	1
0	0	1
0	1	0
0	2	1
0	1	0
0	1	1
0	2	1
0	1	1
0	2	1
0	1	1
0	1	1
0	0	0
0	1	1
0	1	0
0	1	1
0	0	0
0	1	1
0	0	1
0	2	1
0	2	0
0	1	1
0	1	1
0	0	0
0	1	1
0	1	1
0	2	1
0	1	1
0	0	0
0	0	1
0	0	1
0	3	1
0	1	1
0	2	1
0	1	1

0	1	0
0	0	1
0	0	1
0	1	1
0	0	1
0	0	1
0	1	1
0	2	0
0	0	1
0	1	1
0	0	0
0	1	1
0	0	0
0	1	1
0	0	1
0	2	1
0	0	0
0	1	1
0	3	0
0	1	0
0	3	0
0	2	1
0	0	1
0	2	1
0	1	1
0	4	1
0	0	1
0	0	0
0	1	1
0	2	1
0	1	1
0	0	1
0	1	1
0	3	1
0	1	1
0	1	0
0	0	1
0	0	1
0	0	1
0	1	1
0	1	1
0	0	0
0	0	0

0	1	1
0	1	0
0	0	1
0	0	0
0	1	1
0	0	1
0	0	1
0	0	1
0	3	1
0	1	1
0	2	1
0	0	1
0	1	1
0	0	0
0	0	1
0	1	1
0	1	0
0	0	0
0	0	1
0	1	1
0	0	0
0	1	1
0	0	1
0	2	1
0	0	1
0		1
0	1	1
0	0	1
0	2	1
0	1	0
0	0	0
0	0	0
0	0	1
0	0	1
0	0	1
0	1	1
0	0	1
0	2	1
0	2	1
0	0	0
0	0	1
0	1	1
0	1	1

0	0	1
0	1	1
0	4	1
0	1	1
0	0	1
0	0	1
0	1	1
0	1	1
0	1	0
0	3	1
0	1	1
0	2	1
0	0	1
0	1	1
0	0	1
0	0	1
0		1
0	1	1
0	0	0
0	0	0
0	1	1
0	0	1
0	0	1
0	2	0
0	1	1
0	0	1
0	0	1
0	0	1
0	0	1
0	4	0
0	1	1
0	1	1
0	2	1
0	2	1
0	2	1
0	1	0
0	2	1
0	1	1
0	3	1
0	1	1
0	2	1
0	1	1
0	1	1

0	0	1
0	0	0
0	1	1
0	3	1
0	3	1
0	0	1
0	0	1
0	0	1
0	0	0
0	2	1
0	0	1
0	1	1
0	0	1
0	0	1
0	0	0
0	0	0
0	1	1
0	0	1
0	0	1
0	0	1
0	0	1
0	0	0
0	3	1
0	0	1
0	0	1
0	0	1
0	0	1
0	0	1
0	0	1
0	1	1
0	1	1
0	0	1
0	0	1
0	0	1
0	0	1
0	0	1
0	1	1
0	0	1
0	1	1
0	2	1
0	0	1
0	4	1
0	0	1

1	5	1
0	1	1
0	2	1
0	0	1
0	5	1
0	0	1
0	3	1
0	0	1
0	0	1
0	0	1
0	0	0
0	0	1
0	0	1
0	0	1
0	0	0
0	4	1
0	0	1
0	0	1
0	0	1
0	0	0
0	1	1
0	1	1
0	0	1
0	1	1
0	3	1
0	0	1
0	1	1
0	4	1
0	0	0
0	3	1
0	0	1
0	3	0
0	0	1
0	1	1
0	0	1
0	1	1
0	0	1
0	3	1
0	1	1
0	0	1
0	0	1
0	1	1
0	0	1

0	3	0
0	0	1
0	3	1
0	0	1
0	0	1
0	0	1
0	0	1
0	3	0
0	0	1
0	3	1
0	0	1
0	3	0
0	0	1
0	1	1
0	5	0
0	0	1
0	1	1
0	0	1
0	3	1
0	0	1
0	0	1
0	0	0
0	0	1
0	1	0
0	0	1
	0	0
0	1	1
0	0	1
0	0	0
0	3	1
0	1	1
0	2	1
0	0	0
0	0	0
0	1	0
0	2	1
0	0	0
0	0	1
0	4	1
0	0	1
0	0	1
0	3	1
0	1	1

0	3	1
0	0	1
0	0	1
0	1	1
0	0	1
0	3	1
0	4	1
0	1	1
0	1	1
0	0	1
0	0	1
0	3	1
0	3	1
0	1	1
0	0	1
0	1	1
0	1	1
0	2	1
0	3	1
0	0	1
0	0	1
0	0	1
0	0	1
0	0	1
0	0	1
0	0	1
0	0	1
0	2	1
0	3	1
0	0	1
0	1	1
0	2	1
0	0	0
0	0	0
0	0	1
0	1	0
0	1	1
0	4	1
0	4	1
0	0	1
0	2	1
0	0	1
0	1	1

0	2	1
0	0	1
0	2	1
0	0	1
0	1	1
0	0	1
0	4	1
0	2	1
0	1	1
0	0	1
0	4	1
0	2	1
0	1	1
0	4	1
0	0	0
0	1	1
0	1	0
0	1	1
0	0	1
0	3	1
0	2	1
0	1	1
0	0	1
0	0	1
0	2	1
0	2	1
0	3	1
0	0	1
0	0	1
0	2	0
0	1	1
0	1	1
0	2	0
0	0	1
0	0	1
0	3	1
0	0	1
0	0	1
0	0	0
0	1	1
0	0	0
0	0	1
0	1	1

0	0	0
0	0	1
0	1	0
0	2	1
0	0	1
0	1	1
0	0	1
0	0	1
0	0	1
0	0	1
0	0	1
0	0	1
0	1	1
0	0	1
0	2	1
0	1	0
0	3	1
0	0	1
0	4	1
0	0	1
0	1	1
0	0	1
0	3	0
0		1
0	0	1
0	0	1
0	3	1
0	3	1
0	1	1
0	0	1
0	0	0
0	4	1
0	0	1
0	0	0
0	2	1
0	3	1
0	0	1
0	4	1
0	2	1
0	0	1
0	0	1
0	0	1
0	0	1

0	0	0
0	0	1
0	1	0
0	0	1
0	2	0
0	0	1
0	0	1
0	1	1
0	1	1
0	1	1
0	3	1
0	0	1
0	1	1
0	1	1
0	0	1
0	3	1
0	0	1
0	0	1
0	1	1
0	1	1
0	0	1
0	1	1
0	1	1
0	2	1
0	3	1
0	6	1
0	0	1
0	3	0
0	0	1
0	0	1
0	1	1
0	1	0
0	2	1
0	4	1
0	0	1
0	0	1
0	0	1
0	2	1
0	2	1
0	0	1
0	0	1
0	0	1
0	1	1

0	0	1
0	1	1
0	0	1
0	0	1
0	0	1
0	2	1
0	0	0
0	5	0
0	0	1
0	0	1
0	0	1
0	3	1
0	0	1
0	0	0
0	0	1
0	2	1
0	0	1
0	3	1
0	0	1
0	0	1
0	0	1
0	0	1
0	0	0
0	2	1
0	2	0
0	3	0
0	1	1
0	0	1
0	1	1
0	0	1
0	0	1
0	1	1
0	1	1
0	1	0
0	0	1
0	2	1
0	3	1
0	0	1
0	0	1
0	0	1
0	0	1
0	0	1
0	0	1

0	4	1
0	0	1
0	0	1
0	3	1
0	1	0
0	0	0
0	1	1
0	0	1
0	0	1
0	1	1
0	0	1
0	0	1
0	0	1
0	0	1
0	0	1
0	0	1
0	0	1
0	2	1
0	0	1
0	1	1
0	0	1
0	1	1
0	1	1
0	0	1
0	0	1
0	1	1
0	0	1
0	3	1
0	0	1
0	6	1
0	2	1
0	0	1
0	1	0
0	1	1
0	0	0
0	0	0
0	2	1
0	0	1
0	2	1
0	3	1
0	0	1
0	0	1
0	0	0

0	1	1
0	3	1
0	1	1
0	0	1
0	1	1
0	0	1
0	0	0
0	0	0
0	0	1
0	0	1
0	0	1
0	0	1
0	0	0
0	0	1
0	0	0
0	3	1
0	1	1
0	0	1
0	0	1
0	1	1
0	1	1
0	2	1
0	0	1
0	1	1
0	1	1
0	2	1
0	0	1
0	4	1
0	5	1
0	0	1
0	1	1
0	1	1
0	0	1
0	3	1
0	0	1
0	0	1
0	0	1
0	3	1
0	0	1
0	0	1
0	1	0
0	0	1
0	2	1

0	3	1
0	1	1
0	0	1
0	0	1
0	4	1
0	2	1
0	0	1
0	0	0
0	0	1
0	0	0
0	0	1
0	0	1
0	0	1
0	1	1
0	0	1
0	0	1
0	0	1
0	3	1
0	2	1
0	3	1
0	1	1
0	0	0
0	4	1
0	0	1
0	4	1
0	0	1
0	3	1
0	0	1
0	2	1
0	1	1
0	3	1
0	4	1
0	0	1
0	3	1
0	5	0
0	2	1
0	4	1
0	3	1
0	2	1
0	2	1
0	0	1
0	0	1
0	0	0

0	0	1
0	3	1
0	2	1
0	0	1
0	1	1
0	0	1
0	0	1
0	0	1
0	0	1
0	0	1
0	0	1
0	0	1
0	0	1
0	0	1
0	0	1
0	0	0
0	3	1
0	0	0
0	2	1
0	1	1
0	2	1
0	1	1
0	3	1
0	2	1
0	0	1
0	0	1
0	0	1
0	2	1
0	2	1
0	1	1
0	2	1
0	0	1
0	2	1
0	0	1
0	0	1
0	6	0
0	1	1
0	2	1
0	1	1
0	0	0
0	0	1
0	1	1
0	3	1

0	2	1
0	3	0
0	0	1
0	3	0
0	0	1
0	1	1
0	0	1
0	0	0
0	1	1
0	0	1
0	0	1
0	0	1
0	0	1
0	1	1
0	3	1
0	1	1
0	0	0
0	0	1
0	1	0
0	0	1
0	0	0
0	0	1
0	0	1
0	1	1
0	2	1
0	1	1
0	2	1
0	2	1
0	0	1
0	0	1
0	5	1
0	2	1
0	0	1
0	0	1
0	1	1
